# Chloride nutrition improves drought resistance by enhancing water deficit avoidance and tolerance mechanisms

**DOI:** 10.1093/jxb/erab143

**Published:** 2021-03-30

**Authors:** Juan D Franco-Navarro, Pablo Díaz-Rueda, Carlos M Rivero-Núñez, Javier Brumós, Alfredo E Rubio-Casal, Alfonso de Cires, José M Colmenero-Flores, Miguel A Rosales

**Affiliations:** 1 Group of Plant Ion and Water Regulation, Instituto de Recursos Naturales y Agrobiología, Consejo Superior de Investigaciones Científicas (CSIC), Seville, Spain; 2 Instituto Valenciano de Investigaciones Agrarias, Centro de Genómica, Moncada, Valencia, Spain; 3 Departamento de Biología Vegetal y Ecología, Facultad de Biología, Universidad de Sevilla, Seville, Spain; 4 Laboratory of Plant Molecular Ecophysiology, Instituto de Recursos Naturales y Agrobiología, Consejo Superior de Investigaciones Científicas (CSIC), Seville, Spain; 5 Lancaster University, UK

**Keywords:** Beneficial macronutrient, chloride, drought resistance, photosynthesis, turgor, water deficit, water relations, WUE

## Abstract

Chloride (Cl^−^), traditionally considered harmful for agriculture, has recently been defined as a beneficial macronutrient with specific roles that result in more efficient use of water (WUE), nitrogen (NUE), and CO_2_ in well-watered plants. When supplied in a beneficial range of 1–5 mM, Cl^−^ increases leaf cell size, improves leaf osmoregulation, and reduces water consumption without impairing photosynthetic efficiency, resulting in overall higher WUE. Thus, adequate management of Cl^−^ nutrition arises as a potential strategy to increase the ability of plants to withstand water deficit. To study the relationship between Cl^−^ nutrition and drought resistance, tobacco plants treated with 0.5–5 mM Cl^−^ salts were subjected to sustained water deficit (WD; 60% field capacity) and water deprivation/rehydration treatments, in comparison with plants treated with equivalent concentrations of nitrate, sulfate, and phosphate salts. The results showed that Cl^−^ application reduced stress symptoms and improved plant growth during water deficit. Drought resistance promoted by Cl^−^ nutrition resulted from the simultaneous occurrence of water deficit avoidance and tolerance mechanisms, which improved leaf turgor, water balance, photosynthesis performance, and WUE. Thus, it is proposed that beneficial Cl^−^ levels increase the ability of crops to withstand drought, promoting a more sustainable and resilient agriculture.

## Teaser

Chloride nutrition at macronutrient levels reduces stress symptoms and enhances plant growth during water deficit, promoting water deficit avoidance (water-use efficiency) and tolerance (osmoregulation) mechanisms that improve drought resistance.

## Introduction

In the context of the climate change and strong water demand from intensive agriculture, drought is certainly the abiotic stress that most severely affects crop productivity (Comas *et al.*, 2013; FAO, 2016). Understanding how plants respond to water availability and how water is used for optimal biomass production and yield has gained enormous importance in agriculture (Davies and Bennett, 2015; Maurel and Nacry, 2020). In general, lower availability of soil water during drought leads to a decrease of leaf relative water content (RWC) and leaf water potential (Ψ _w_) that causes abscisic acid (ABA) biosynthesis (McAdam and Brodribb, 2016, 2018; Sack *et al.*, 2018), triggering complex plant acclimatization responses at molecular, cellular, and physiological levels. These responses include water deficit (WD) avoidance and tolerance mechanisms, according to the nomenclature of Levitt (1972). Avoidance responses include mechanisms that maintain plant water content and Ψ_w_ close to unstressed levels, mainly by increasing water uptake or limiting water loss. Induction of stomatal closure reduces water loss through transpiration (Rosales *et al.*, 2012; Koevoets *et al.*, 2016; Buckley, 2019), but leads to a reduction of CO_2_ availability and photosynthesis and, consequently, to the decrease of vegetative growth and yield (Galmés *et al.*, 2007; Chaves *et al.*, 2009; Ferguson *et al.*, 2018). In addition, mechanisms that improve soil water uptake are also stimulated (Rosales *et al.*, 2019; Scharwies and Dinneny, 2019). When WD avoidance mechanisms are overcome and plant tissues experience cellular dehydration, tolerance mechanisms must ensure cell survival and the plant ability to resume growth, including the induction of cell osmotic adjustment and the biosynthesis of protective solutes and proteins (Verslues *et al.*, 2006).

Considering that 80% of the available freshwater resources are currently consumed by agriculture, the improvement of water-use efficiency (WUE), defined as the amount of carbon fixed in photosynthesis per unit of water transpired, remains essential for establishing a balance between agriculture and water resources (Condon *et al.*, 2004; Flexas *et al.*, 2016). Because of the urgent need to improve the world’s crop production, WUE is considered an essential trait to minimize the loss of water in plants. As a consequence, considerable efforts have been made to elucidate physiological and genetic factors associated with this trait (Condon *et al.*, 2004; Blum, 2009; Hessini *et al.*, 2009; Medrano *et al.*, 2015). Several strategies have focused on obtaining new crop varieties with higher WUE and on better management of water resources, such as: (i) improving the irrigation processes and reducing the water loss through soil evaporation or leakage; (ii) increasing the efficiency of fixing carbon in relation to water transpired; and (iii) partitioning more of the achieved biomass into the harvested product (reviewed in Condon *et al.*, 2004). However, due to the complexity of these traits, simpler and more specific aspects of WUE are required to identify single targets of manipulation (Flexas *et al.*, 2016).

Chloride (Cl^−^) has been well characterized as a micronutrient, playing an essential role as a cofactor for PSII and regulating the activity of some enzymes (Broadley *et al.*, 2012). In addition, Cl^−^ is a major osmotically active solute in the vacuole (Flowers, 1988). As a counter anion, Cl^−^ plays relevant roles in regulating the electrical potential of different membranes, the organellar pH gradients, and the electrical excitability of plant cells (White and Broadley, 2001). However, Cl^−^ has been traditionally considered harmful for agriculture, for two main reasons: (i) the toxicity resulting from excessive Cl^−^ accumulation in sensitive crops under salt stress conditions (Li *et al.*, 2017; Geilfus, 2018); and (ii) the generalized belief that Cl^−^ antagonizes nitrate (NO_3_^−^) homeostasis, impairing the ability of crops to transport and accumulate NO_3_^−^ (Kafkafi *et al.*, 1982; Siddiqi *et al.*, 1990; Xu *et al.*, 2000; Wege *et al.*, 2017). However, Cl^−^ nutrition to typical macronutrient levels has been recently uncovered as beneficial for plant growth under well-watered conditions, with new biological functions that improve cell water balance, whole-plant water relations, photosynthesis performance, WUE, and nitrogen-use efficiency (NUE; i.e. the vegetative or reproductive biomass yield per unit of nitrogen available in the soil) in plants (Franco-Navarro *et al*., 2016, 2019; Rosales *et al.*, 2020). Thus, Cl^−^ has been proposed as a beneficial macronutrient (Franco-Navarro *et al.*, 2016), a definition further supported by others (Raven, 2017; Wege *et al.*, 2017; Geilfus, 2018; Orieux *et al.*, 2018; Bazihizina *et al.*, 2019; Raven, 2020). Firstly, when supplied above the micronutrient requirement and below the toxicity threshold (e.g. 1–5 mM Cl^−^), Cl^−^ plays specific roles in the regulation of cell osmolarity and turgor, stimulating leaf cell size and leaf water balance. The resulting enlargement of leaf cell size reduces the stomatal density, which in turn lowers stomatal conductance (*g*_s_) and water consumption. Secondly, Cl^−^ also increases mesophyll diffusion conductance to CO_2_ (*g*_m_), which makes it possible to maintain the plant photosynthetic capacity despite the reduction of *g*_s_, resulting in overall higher WUE in well-watered plants (Franco-Navarro *et al.*, 2019). Therefore, adequate management of Cl^−^ nutrition to improve crop yield while also reducing water consumption is particularly challenging in C_3_ plants (Maron *et al.*, 2019).

Cl^−^ fluxes are also relevant for adequate regulation of stomatal closure (Nieves-Cordones *et al.*, 2019) and specifically required for cell osmotic adjustment in response to osmotic stress (Shabala and Lew, 2002). Therefore, through its role in the regulation of cell osmolarity, water balance, and WUE under well-watered conditions, Cl^−^ homeostasis arises as a potential adaptive mechanism that might increase the ability of plants to withstand drought stress. So far, all previously reported functions of Cl^−^ nutrition as a beneficial macronutrient have been experimentally performed under well-watered conditions. No direct relationship between Cl^−^ and drought resistance in glycophyte plants has been established to date. Therefore, the aim of this work is to elucidate this question by: (i) quantifying the degree of WD resistance of Cl^−^-treated plants compared with plants treated with equivalent concentrations of anionic macronutrients such us NO_3_^−^, phosphate, and sulfate; and (ii) identifying relevant physiological mechanisms regulated by Cl^−^ nutrition that improve WD resistance in plants.

## Materials and methods

### Plant cultivation and experimental design

Tobacco (*Nicotiana tabacum* L. var. Havana) plants were grown under greenhouse experimental conditions (temperature of 25/17±2 °C day/night, relative humidity of 60±10%, and a 16 h/8 h photoperiod with a photosynthetic photon flux density of 300–350 μmol m^−2^ s^−1^). Plants were grown in 7.5 liter pots (20×17×25 cm) containing a mix of perlite:vermiculite (4:6), and watered with a basal nutrient solution supplemented with three nutritional treatments: 5 mM Cl^−^ salts (CL), 5 mM NO_3_^−^ salts (N), and a mix of sulfate+phosphate (SO_4_^2−^+PO_4_^3−^) salts (SP), as previously reported in Franco-Navarro *et al.* (2016). The CL treatment was performed with the application of 5 mM Cl^−^: 2.5 mM KCl, 0.625 mM MgCl_2_, and 0.625 mM CaCl_2_. To evaluate the specificity of Cl^−^ in the studied phenomena, two additional treatments were used: N treatment containing 2.5 mM KNO_3_, 0.625 mM Mg(NO_3_)_2_, and 0.625 mM Ca(NO_3_)_2_; and SP treatment containing 1.25 mM KH_2_PO_4_, 0.625 mM K_2_SO_4_, 0.625 mM MgSO_4_, and 0.625 mM CaSO_4_. All treatments (CL, N, and SP) contained the same cationic balance as shown in Franco-Navarro *et al.* (2016). Nutrients present in the basal nutrient solution were as follows: 1.25 mM KNO_3_, 0.625 mM KH_2_PO_4_, 0.053 mM K_2_HPO_4_, 2 mM Ca(NO_3_)_2_, 1 mM MgSO_4_, 0.1 mM FeNa-EDTA, 0.1 mM H_3_BO_3_, 0.1 mM MnSO_4_, 29 μM ZnSO_4_, 0.11 μM CoCl_2_, 53 μM KCl, 0.1 μM CuSO_4_, 1 μM Na_2_MoO_4_, and 5 μM KI. All experimental solutions were adjusted to pH 5.7 with KOH.

After 30 d (45 days after sowing; DAS), in addition to the three nutritional treatments, plants were subjected to two irrigation treatments: optimal irrigation (control; CTR), in which pots containing tobacco plants were irrigated up to 100% field capacity (3.5 ml g^−1^ substrate) throughout the experiment, and WD, with pots irrigated every 2–3 d up to 60% of field capacity (2.1 ml g^−1^ substrate) for 20 d (65 DAS). During the WD treatment, the resulting average soil water content ranged between 60% and 10% of field capacity (Supplementary Fig. S1A).

Another set of experiments with increasing concentrations of Cl^−^ and SO_4_^2−^+PO_4_^3−^ salts, in combination with CTR and WD regimes (100% and 60% of field capacity, respectively) as explained above, was performed for 26 d under similar experimental conditions to those previously described (Supplementary Fig. S1B). For CL treatments, 0.5, 2, and 5 mM Cl^−^ salts were applied to the basal solution, whereas the equivalent SP treatments were also added to ensure the same cationic balance as in different CL treatments (as described in Franco-Navarro *et al.*, 2016).

### Plant sampling and determination of biomass and leaf parameters

Samplings were performed from each combination of nutritional and irrigation treatments after 20 d or 26 d of water restriction, in which all plants were non-senescent and at the early reproductive stage. Different plant tissues were harvested separately and leaf area was measured as explained below. Subsequently, FW values from different plant tissues were obtained, and samples were dried in a forced-air oven at 75 °C for 48 h to obtain the DW values, both parameters recorded as grams per plant.

After obtaining FW values, detached leaves of each tobacco plant were photographed and their leaf area was measured through pixel quantification with ImageJ2 Software with a high precision of 99.95–100% (Rasband, 1997; Rueden *et al.*, 2017). Data were obtained in cm^2^. Specific leaf area (SLA) was calculated as follows (Marcelis *et al.*, 1998): SLA=(total leaf area)(total leaf DW)^−1^.

### Nutrient content determination

Oven-dried leaf tissue was ground to powder using a homogenizer (Taurus, 25790, Barcelona, Spain) and the concentration of Cl^−^, NO_3_^−^, SO_4_^2−^, and PO_4_^3−^ was determined as previously reported (Franco-Navarro *et al.*, 2016).

### Water parameters

Leaf water content, RWC, succulence, leaf osmotic potential (Ψ _π_), leaf Ψ _w_, and leaf turgor (or pressure) potential (Ψ _p_) were determined as previously described in Franco-Navarro *et al.* (2016).

Water consumption was quantified gravimetrically by recording the weight loss of each pot, equivalent to the volume of solution consumed and lost by evapotranspiration by each plant. In WD-treated plants, water consumption was quantified as the volume of water needed to maintain field capacity up to 60%. Integrated WUE (WUE_i_) was calculated as the increase of plant DW over time related to the accumulated water consumption (g DW ml^−1^ H_2_O), as well as the DW obtained throughout the experiment and after harvesting related to total water consumption (g DW ml^−1^ H_2_O) (Abbate *et al.*, 2004).

### Water deprivation and rehydration assay: quantum yield and pressure probes

Six tobacco plants of each nutritional treatment (SP, CL, and N) were maintained under CTR conditions up to 73 DAS, when water deprivation was applied for 4 d, and, at 77 DAS, plants were rehydrated at 100% of field capacity and monitored until 80 DAS. Three plants from each nutritional treatment were monitored every day by gravimetric methods to verify the water content in the soil, and PSII quantum yield (Qy) measurements were performed. For Qy determination, chlorophyll fluorescence in light-adapted plants was measured using a portable fluorometer (FluorPen FP-100; Photon System Instruments, Brno, Czech Republic), as described in Franco-Navarro *et al.* (2016). For each treatment, 3–5 photosynthetically active and fully expanded intermediate leaves from six plants were used. Qy measurements were conducted every day between 10 h and 12 h from the beginning of the water restriction treatment (46–64 DAS).

For the other three plants, each plant was monitored with 2–3 LPCP probes (so-called ZIM probes; ZIM Plant Technology GmbH, Hennigsdorf, Germany), a non-invasive technique that records leaf turgor pressure in real-time (described in detail in Zimmermann *et al*., 2008, 2010). The leaf patch output pressure (*P*_p_) is recorded in a leaf that is patched between a metallic sensing chip and a magnetic pad. *P*_p_ is inversely correlated with the leaf turgor pressure (Ehrenberger *et al.*, 2012). Signals are sent wirelessly by transmitters to a controller that transfers the data to a GPRS modem linked to an Internet server. Probes and the Internet-based data transfer system were purchased from ZIM Plant Technology GmbH. Probes were clamped on 2–3 photosynthetically active and fully expanded intermediate leaves (fifth–sixth leaves from the top of the plant, at ~0.80 m above the ground), between the central vascular bundle and the edge of the leaves (~3 cm away from the edge), and in the middle part of those leaves, in order to establish a uniform contact with the leaf tissue avoiding nerves (Fernández *et al.*, 2011). The clamping was performed pre-dawn at maximum turgidity as recommended by Zimmermann *et al*. (2008, 2010, 2013). Pressure signals were appropriately adjusted between 10 kPa and 25 kPa, changing the distance between the two magnets. The pressure sensor magnet was placed on the abaxial side of the leaves. When pressure probe recordings became stable at 68 DAS, variation in the diurnal amplitude of *P*_p_ was found because of possible differences in the initial clamp pressure, leaf thickness, or compressibility variations as reported in Zimmermann *et al.* (2008).

### Leaf gas exchange parameters

Net photosynthetic rate (*A*_N_) and stomatal conductance (*g*_s_) were measured between 12.00 h and 14.00 h using an open gas exchange system (LI-6400, LI-COR, Lincoln, NE, USA) equipped with a 2×3 cm LED chamber (LI-6400-02B) as described in Franco-Navarro *et al.* (2016). The WUE_i_ was calculated as the ratio between the rate of photosynthesis and stomatal conductance (*A*_N_/*g*_s_).

### Statistical analyses

Statistical analyses were performed using STATGRAPHICS Centurion XVI software (StatPoint Technologies, Warrenton, VA, USA). Shapiro–Wilk (W) test was used to verify the normality of the data sets. One-way ANOVA and multivariate analysis of variance (MANOVA) were performed to determine significant differences between groups of samples, and levels of significance were described by asterisks: *P≤0.05; **P≤0.01; ***P≤0.001. Non-significant (ns) differences were indicated when *P* was >0.05. Multiple comparisons of means were determined by the Tukey’s HSD (honestly significant difference) and MRT (multiple range test) statistical tests included in the mentioned software. Analysis of covariance (ANCOVA) was performed with R software (https://www.r-project.org/) to compare the slopes of the relationship of total biomass with water consumption between CL and SP treatments. Values represent the mean of at least six tobacco plants in each treatment, which were reproduced in at least three independent experiments (Supplementary Table S1).

## Results

### The effect of Cl^−^ on plant growth during water deficit

To study whether Cl^−^ nutrition participates in plant adaptive responses to drought stress, greenhouse experiments were performed under two irrigation regimes: optimal irrigation (CTR) and sustained water deficit (WD). For the WD treatment, plants were watered every 2–3 d with the three nutritional treatments (CL, N, and SP) until the substrate reached 2.1 ml g^−1^ (60% of field capacity). The WD treatment was maintained for 20 d, whereas watering up to 100% of field capacity was established for the CTR treatment (3.5 ml g^−1^). The time course of the substrate water loss throughout a representative experiment is presented in Supplementary Fig. S1A.

First, we verified whether the effects of the 5 mM Cl^−^ treatment (CL) on plant growth were consistent with those previously obtained in Franco-Navarro *et al*. (2016, 2019). With this aim, we conducted new sets of experiments (Supplementary Table S1) and compared different nutritional and physiological effects of the CL treatment with those of plants subjected to low Cl^−^ (SP and N treatments). Consistently, leaf anion contents (Cl^−^, NO_3_^−^, SO_4_^2−^, and PO_4_^3−^) were differentially accumulated in plants according to the respective nutritional treatments (CL, N, and SP) under both irrigation regimes (Table 1; Supplementary Table S2). Nutritional and irrigation treatments and their interaction significantly affected Cl^−^ and NO_3_^−^ contents in tobacco leaves (Table 1; Supplementary Table S2). Under control conditions, the Cl^−^ concentration in CL-treated leaves was 106.5 mM (i.e. 55.7 mg g^−1^ DW), reaching typical macronutrient levels. In SP and N plants, Cl^−^ content was 100 times lower, although far exceeding the critical levels of deficiency required to fulfil essential micronutrient functions (Broadley *et al.*, 2012; Colmenero-Flores *et al.*, 2019). Interestingly, the Cl^‒^ content significantly increased in drought-stressed CL and SP plants (1.12 and 2.5 times, respectively), whereas no changes were observed in N plants. In addition, the NO_3_^−^ concentration was strongly decreased by WD in SP and N plants (2.1 and 3.3 times, respectively), whereas no relevant changes in SO_4_^2−^ and PO_4_^3−^ contents were observed (Table 1).

**Table 1.** Anion concentration in leaves subjected to different nutritional and irrigation treatments

	Cl^−^ (mM)			NO_3_^−^ (mM)			PO_4_^3−^ (mM)			SO_4_^2−^ (mM)		
	CTR	WD	*P*	CTR	WD	*P*	CTR	WD	*P*	CTR	WD	*P*
**SP**	1.02±0.08 b	2.56±0.61 b	*	6.71±1.18 b	3.20±0.78 b	*	15.7±1.51 a	13.8±1.62 a	ns	33.5±1.80 a	30.8±4.06 a	ns
**CL**	106.5±3.85 a	118.9±2.50 a	*	2.48±0.28 b	2.00±0.42 b	ns	6.97±0.58 b	8.76±0.29 b	**	12.0±2.55 b	11.1±0.38 b	ns
**N**	1.01±0.16 b	1.05±0.10 b	ns	46.9±7.22 a	14.2±1.19 a	**	9.02±0.28 b	9.01±0.24 b	ns	15.5±3.56 b	17.2±1.20 b	ns
***P***	***	***		***	***		***	***		***	***	
**I**	**			***			ns			ns		
**NT**	***			***			***			***		
**I×NT**	**			***			ns			ns		

Nutritional treatment (NT) consisted of a basal nutrient solution supplemented with 5 mM chloride (CL), 5 mM nitrate (N), or the sulfate+phosphate (SP) salt mixture containing the same cationic balance as in the CL and N treatments. Irrigation treatment (I) consisted of a control treatment of well-watered plants (CTR; 100% field capacity) and sustained water deficit (WD; 60% field capacity) treatments. Mean values ±SE, *n*=6. Levels of significance: ***P≤0.001,**P≤0.01, *P≤0.05, and *P* >0.05 (‘ns’, non-significant). ‘Homogeneous group’ statistics were calculated through ANOVA and MANOVA tests, where mean values with different letters are significantly different according to Tukey’s test.

**Fig. 1.**
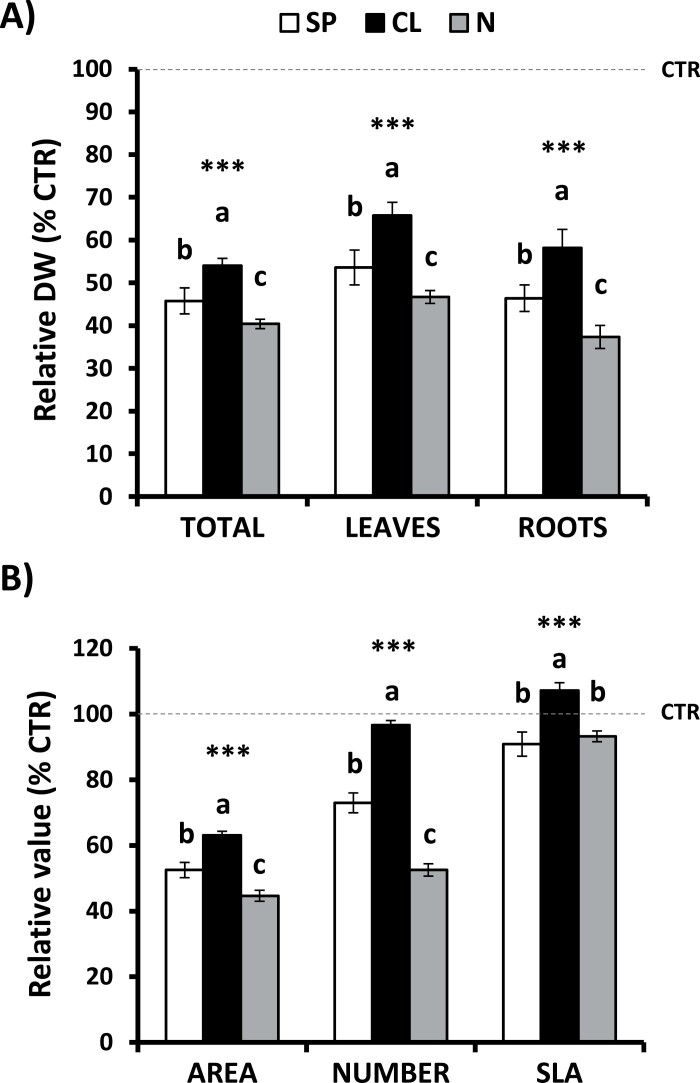
Effect of Cl^−^ nutrition and sustained water deficit on plant growth. Plants were alternatively treated with (i) three nutritional treatments: 5 mM chloride salts (CL), 5 mM nitrate salts (N), and a mixture of sulfate+phosphate salts (SP) containing the same cationic balance as in the CL and N treatments; and (ii) two irrigation treatments: 100% field capacity (CTR, control) and 60% field capacity (WD, water deficit). (A) Effect on total, leaf, and root DW (%) in WD plants normalized to CTR plants. (B) Effect on leaf area and number and specific leaf area (SLA) in WD plants normalized to CTR plants. Absolute values of CTR treatments were as follows: total DW (g), SP=23.2±1.19, CL=27.9±0.58, N=43.8±1.66; leaf DW (g), SP=7.60±0.34, CL=9.43±0.40, N=16.4±0.63; root DW (g), SP=2.47±0.16, CL=2.96±0.13, N=4.82±0.25; leaf area (cm^2^), SP=2156±44.5, CL=2575±49.7, N=4198±75.1; number of leaves, SP=17.8±0.47, CL=14.3±0.43, N=26.5±0.73; SLA (cm^2^ g^-1^ DW), SP=273.5±6.72, CL=269.4±1.53, N=263.1±4.45. Mean values ±SE. *n*=6. ‘Homogeneous group’ statistics were calculated through ANOVA, where mean values with different letters are significantly different according to Tukey’s test at *P*≤0.05. Levels of significance: ***P≤0.001.

As demonstrated in Franco-Navarro *et al.* (2016), the application of 5 mM Cl^−^ under control conditions promoted plant growth when compared with SP plants (Supplementary Figs S2, S3A–C), mainly due to higher leaf expansion and shoot growth (Supplementary Fig. S3D), which was in turn a consequence of the stimulatory effect of Cl^−^ on cell expansion (Franco-Navarro *et al.*, 2016). On the other hand, the N treatment strongly stimulated plant growth and leaf expansion as a result of a higher rate of both cell division and metabolic activity given the important role of nitrogen in plant metabolism, growth, and development (Hawkesford *et al.*, 2012; Franco-Navarro *et al*., 2016, 2019; Supplementary Figs S2, S3A–C). When evaluating growth responses to drought, we found that plants subjected to WD showed reduced total, leaf, and root biomass under all nutritional treatments, with significant interactions between irrigation and nutritional treatments (Fig. 1A; Supplementary Figs S2, S3A–C). However, the Cl^−^ application caused lower reduction of plant growth (35–45% reductions of total and organs biomass) than SP and N treatments (45–55% and 50–60% reductions, respectively) during WD (Fig. 1A). To further explore the role of Cl^−^ in plant acclimatization to WD, different morphological parameters widely used as key leaf traits were measured: leaf area, number of leaves, and SLA (i.e. the leaf area per unit of biomass invested). Under control conditions, N plants showed the significantly highest leaf area due to the occurrence of larger and more numerous leaves, while CL plants presented higher leaf area than SP plants (Supplementary Fig. S3D, E). However, non-significant differences in SLA between the three nutritional treatments were observed (Supplementary Fig. S3F). The WD treatment caused a strong reduction in both the area and number of leaves in SP and N plants, which was more significant in N-treated plants (Fig. 1B; Supplementary Fig. S3D, E). Interestingly, WD caused no changes in the number of leaves in CL plants, exhibiting a smaller reduction of leaf area in comparison with the SP and N treatments (Fig. 1B). Furthermore, whereas SP and N plants showed a similar SLA reduction under WD, Cl^−^ application significantly increased it (Fig. 1B; Supplementary Fig. S3F). Taken together, our results validate the beneficial effect of Cl^−^ nutrition on plant growth under both well-watered and WD conditions in tobacco plants, whereas N-treated plants exhibited the highest sensitivity to WD.

### The effect of Cl^−^ nutrition on whole-plant water-use efficiency and water balance during water deficit

**Fig. 2.**
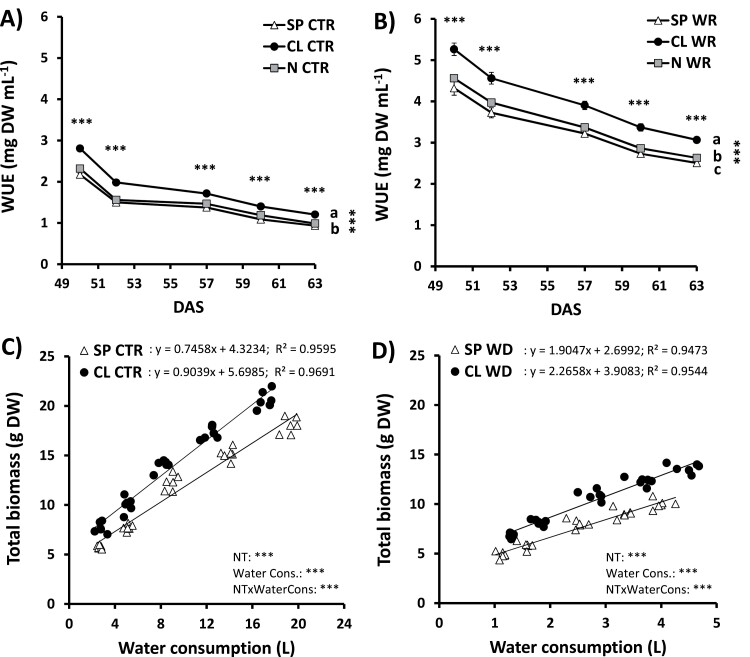
Effect of Cl^−^ nutrition and sustained water deficit on integrated water-use efficiency. Plants were alternatively treated with (i) three nutritional treatments (NT): 5 mM chloride salts (CL); 5 mM nitrate salts (N); and a mixture of sulfate+phosphate salts (SP) containing the same cationic balance as in the CL and N treatments; and (ii) two irrigation treatments were also applied: 100% field capacity (CTR, control) and 60% field capacity (WD, water deficit). Effect on integrated water-use efficiency (WUE) in plants subjected to CTR (A) and WD (B) treatments. Relationship between total biomass and accumulated water consumption in plants during CTR (C) and WD (D) treatments. Mean values ±SE, *n*=6. ‘Homogeneous group’ statistics were calculated through ANOVA and MANOVA, where mean values with different letters are significantly different according to Tukey’s test at *P*≤0.05. The regression line for each SP and CL pool is shown in both panels (C and D), where *P*-values and ANCOVA to compare regression slopes are shown. Levels of significance: ***P≤0.001.

**Fig. 3.**
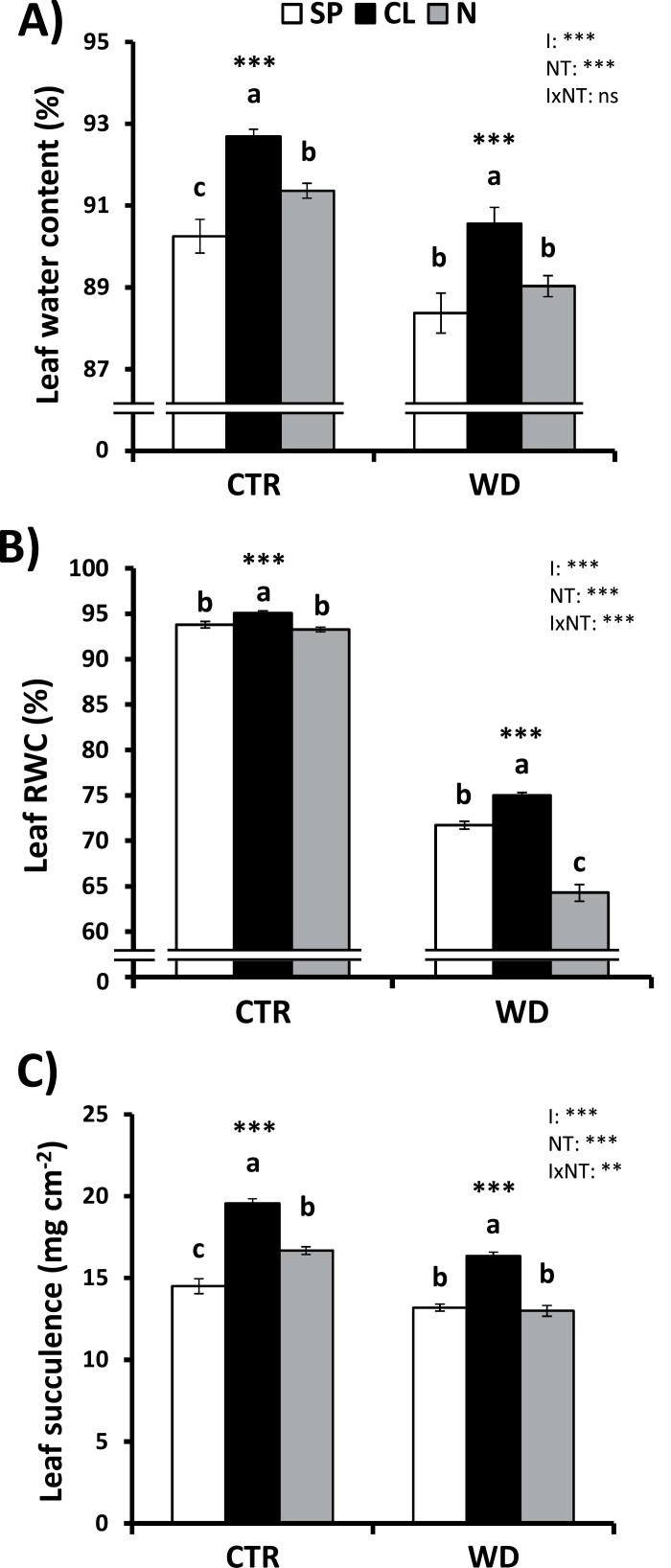
Effect of Cl^−^ nutrition and sustained water deficit on water parameters. Plants were alternatively treated with (i) three nutritional treatments (NT): 5 mM chloride salts (CL), 5 mM nitrate salts (N), and a mixture of sulfate+phosphate salts (SP) containing the same cationic balance as in the CL and N treatments; and (ii) two irrigation treatments (I): 100% field capacity (CTR, control) and 60% field capacity (WD, water deficit). Effect on leaf water content (A), leaf relative water content (RWC) (B), and leaf succulence (C) in CTR and WD treatments. Mean values ±SE, *n*=6. ‘Homogeneous group’ statistics were calculated through ANOVA, where mean values with different letters are significantly different according to Tukey’s test at *P*≤0.05. Levels of significance: ***P≤0.001, **P≤0.01, and ‘ns’ *P*>0.05.

Considering that Cl^−^ nutrition improves whole-plant WUE and water balance in well-watered plants (Franco-Navarro *et al*., 2016, 2019), and alleviates detrimental effects of WD on plant growth (Fig. 1), we wondered whether Cl^−^ nutrition induces plant physiological responses linked to water relations during WD. Measurement of the total plant weight relative to accumulated water consumed showed higher integrated WUE values in Cl^‒^-treated plants during both CTR (Fig. 2A) and WD (Fig. 2B) treatments. Interestingly, when compared with well-watered plants, we observed that WUE values exhibited a >2-fold increase during WD, remaining higher always in CL plants (Fig. 2B). To better compare differences between high and low Cl^−^ treatments, biomass versus water use relationships were plotted in Fig. 2C (CTR) and Fig. 2D (WD). Given that the biomass of N plants differs greatly from that of the other treatments (Supplementary Fig. S3), only the CL versus SP ionic treatments were compared. Using ANCOVA (as reported in Puértolas *et al.*, 2017), significant differences between CL and SP treatments were observed in both CTR and WD treatments, showing that Cl^−^-treated plants have a greater capacity to produce biomass in relation to the amount of water consumed (Fig. 2C, D). It is noteworthy that under control conditions, CL plants maintained higher growth with less water consumed than SP plants (Fig. 2C). However, under WD conditions, CL plants maintained higher WUE (Fig. 2B) despite consuming more water, due to higher biomass production (Fig. 2D). When control and WD values were plotted together (Supplementary Fig. S4), the ANCOVA showed that the slopes of the relationship varied between CL and SP treatments, further supporting WUE differences between ionic treatments. When we delved into leaf-level responses to WD, our results showed that WD reduced leaf water content, RWC, and succulence in plants subjected to all nutritional treatments (Fig. 3). However, these water parameters exhibited a significant Cl^−^-dependent stimulation in comparison with SP and N treatments under both control and WD conditions, showing that Cl^−^ alleviates negative effects of WD on plant water balance.

**Fig. 4.**
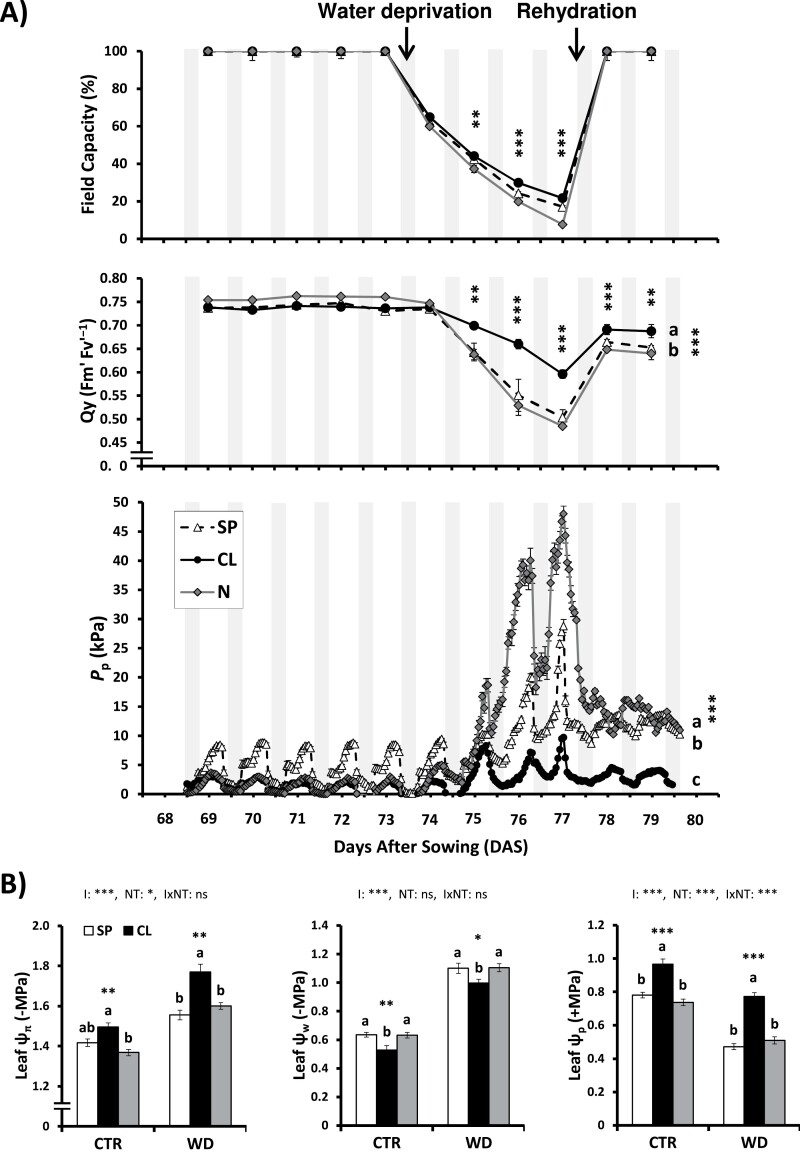
Effect of Cl^−^ nutrition on water status during water deprivation and rehydration treatments. Plants were alternatively treated with three nutritional treatments (NT): 5 mM chloride salts (CL); 5 mM nitrate salts (N); and a mixture of sulfate+phosphate salts (SP) containing the same cationic balance as in the CL and N treatments. Tobacco plants of each NT (SP, CL, and N) were maintained under optimal irrigation (CTR) up to 73 DAS, then water deprivation was applied for 4 d and, at 77 DAS, plants were rehydrated up to 100% of field capacity and further monitored until 80 DAS. (A) Effect on field capacity, efficiency of PSII, and real-time measurement of leaf turgor using the non-invasive magnetic leaf patch-clamp pressure probes (Zimmermann *et al.*, 2008). Patch pressure (*P*_p_) is inversely correlated with leaf turgor pressure and positively correlated with leaf water potential and plant transpiration (Zimmermann *et al*., 2008, 2010). (B) Effect of Cl^−^ nutrition and sustained water deficit on leaf osmotic potential (Ψ _π_), leaf water potential (Ψ _w_), and leaf turgor (or pressure) potential (Ψ _p_) in CL, N, and SP plants, which were treated for 20 d with two irrigation regimes (I): 100% field capacity (CTR, control) and 60% field capacity (WD, water deficit). Mean values ±SE, *n*=6. ‘Homogeneous group’ statistics were calculated through ANOVA and MANOVA, where mean values with different letters are significantly different according to Tukey’s test at *P*≤0.05. Levels of significance: ***P≤0.001; **P≤0.01; and *P≤0.05.

To further investigate the role of Cl^−^ in regulating whole-plant water relations and, particularly, turgor maintenance during water deprivation/rehydration, we monitored the turgor pressure changes of tobacco leaves by using magnetic leaf patch-clamp pressure probes (ZIM-probe; Zimmermann *et al.*, 2008). This non-invasive technique allows the real-time monitoring of the turgor pressure of intact leaves with high precision (Fig. 4A). The measured leaf patch output pressure *P*_p_ is inversely proportional to the leaf turgor. Before water deprivation, *P*_p_ values recorded in the three treatments (SP, CL, and N) gradually increased during the day, indicating turgor loss after sunrise, and abruptly decreased during sunset, indicating leaf turgor recovery during the night. Although some differences in amplitude were found between treatments, the kinetics of the *P*_p_ curves from different plants showed the same circadian trends. Irrigation with the three nutritional treatments (SP, CL, and N) was withheld for 4 d until the water content of drought-stressed pots reached between 10% and 20% of the water content measured in well-watered pots. Subsequently, irrigation was restored to control water levels. After WD imposition, strong loss of turgor (increase in *P*_p_ values) was observed in plants subjected to SP and N treatments. However, turgor values were not significantly altered by WD in CL plants, which maintained a *P*_p_ pattern similar to that of well-watered plants (Fig. 4A). To quantify cell damage produced by the resulting leaf tissue dehydration, the photosynthetic efficiency of PSII was measured with a chlorophyll fluorometer in a dark-adapted state. The CL treatment determined much greater protection of the photosynthetic machinery under severe WD, with significantly higher Qy values than those of SP and N treatments (Fig. 4A). After rehydration, CL plants, but not SP and N plants, fully recovered *P*_p_ and Qy values to those of control conditions.

To determine whether improved water balance parameters of CL plants were associated with the Cl^−^ osmoregulatory properties and the resulting stimulation of leaf turgor observed in well-watered plants (Franco-Navarro *et al.*, 2016; Colmenero-Flores *et al.*, 2019), Ψ _π_, Ψ _w_, and Ψ _p_ were measured in leaves of tobacco plants. Cl^–^-treated plants showed more negative values of Ψ _π_ under both control and WD conditions (Fig. 4B), indicating greater osmoregulatory capacity due to higher accumulation of osmotically active solutes in their leaf tissues. This in turn led to significantly higher Ψ _p_ values in CL plants (Fig. 4B) and, consequently, to a better tolerance to WD. Higher turgor of CL plants was also a consequence of less negative Ψ _w_ values under both control and WD conditions (Fig. 4B), caused by the higher leaf water content of Cl^−^-treated plants (Fig. 3A). Interestingly, the more positive leaf Ψ _w_ of CL plants, in comparison with SP and N plants, indicates that other events affecting plant water relations might be regulated by Cl^−^, as described below.

### The effect of Cl^−^ nutrition on gas exchange and photosynthetic water-use efficiency during water deficit

**Fig. 5.**
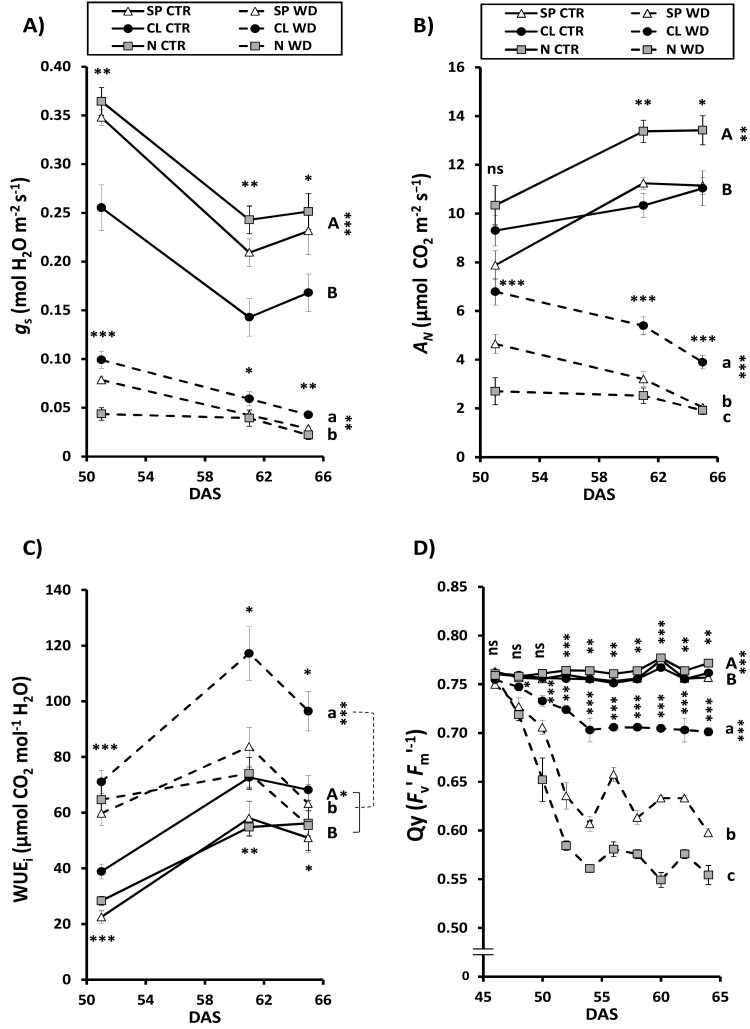
Effect of Cl^−^ nutrition and sustained water deficit on gas exchange parameters, water-use efficiency, and stability of PSII. Plants were alternatively treated with (i) three nutritional treatments (NT): 5 mM chloride salts (CL); 5 mM nitrate salts (N); and a mixture of sulfate+phosphate salts (SP) containing the same cationic balance as in the CL and N treatments; and (ii) two irrigation treatments (I) were also applied: 100% field capacity (CTR, control) and 60% field capacity (WD, water deficit). Effect on (A) stomatal conductance (*g*_s_), (B) net photosynthetic rate (*A*_N_), and (C) photosynthetic or instantaneous water-use efficiency (WUEi) measured in fully expanded photosynthetically active leaves from plants between 51 and 65 days after sowing (DAS). (D) Effect on the highly sensitive physiological stress marker quantum yield (Qy; stability of PSII) measured in fully expanded photosynthetically active leaves from plants between 46 and 65 DAS. Mean values ±SE. *n*=6. ‘Homogeneous group’ statistics were calculated through ANOVA and MANOVA, where mean values with different letters are significantly different according to Tukey’s test at *P*≤0.05. Levels of significance: ***P≤0.001; **P≤0.01; *P≤0.05; and ‘ns’ *P*>0.05.

To better understand the role of Cl^−^ on the regulation of plant water relations, *g*_s_ was quantified under CTR and WD conditions (Fig. 5A). As previously shown (Franco-Navarro *et al.*, 2016), the CL treatment gave rise to lower *g*_s_ in well-watered plants due to the lower stomatal density. The lower *g*_s_ did not impair the net photosynthetic rate when compared with SP plants (*A*_N_; Fig. 5B) as a consequence of the positive effect of Cl^−^ on the *g*_m_ (Franco-Navarro *et al.*, 2019), leading to higher photosynthetic or intrinsic WUE_i_ (*A*_N_/*g*_s_; Fig. 5C). As a result, the better water balance (Fig. 4) and WUE of Cl^−^-treated plants increased their tolerance to WD, as evidenced by the lower cell damage suffered in photosynthetic tissues (Fig. 5D). Therefore, SP and N plants, with more dehydrated and less turgid leaves (Fig. 4B), became more stressed by the WD treatment (Fig. 5D), leading to stronger *g*_s_ reduction (Fig. 5A) and greater loss of photosynthetic capacity (lower *A*_N_; Fig. 5B). Furthermore, the correlation of *A*_N_ versus RWC showed higher *A*_N_ values per unit of RWC in CL plants during WD (Supplementary Fig. S5), demonstrating the greater Cl^−^-induced tolerance to drought stress.

### The dose-dependent effect of Cl^−^ nutrition on water balance and WUE parameters during water deficit

**Fig. 6.**
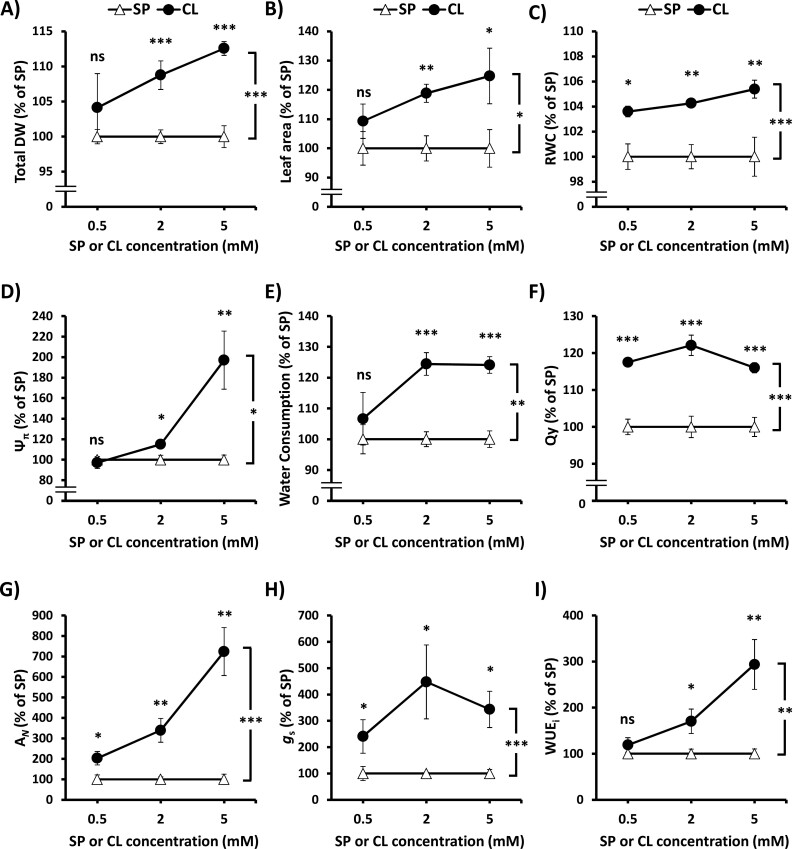
Improvement by Cl^−^ nutrition of plant parameters determining growth, water relations, photosynthesis, and water-use efficiency during water deficit. Plants were alternatively treated with increasing chloride (CL) or sulfate+phosphate (SP) treatments (0.5, 2, and 5 mM), maintaining the same cationic balance. Plants were harvested after 20 d of sustained water deficit (WD; 60% field capacity), and figures show values of CL plants normalized to SP plants, the latter representing 100%. Plant parameters and absolute values of CTR treatments were as follows: (A) total DW (%), 0.5SP=31.2±0.63, 2SP=32.8±2.35, 5SP=33.6±1.41, 0.5CL=32.6±0.52, 2CL=37.3±1.75, 5CL=39.9±1.63; (B) leaf area (%), 0.5SP=2292±146.4, 2SP=2462±147.0, 5SP=2336±118.6, 0.5CL=2388±208.0, 2CL=2657±98.5, 5CL=2845±70.7; (C) relative water content (RWC; %): 0.5SP=93.4±1.1, 2SP=93.6±1.2, 5SP=93.9±0.8, 0.5CL=94.2±0.3, 2CL=95.9±1.7, 5CL=96.2±0.8; (D) leaf osmotic potential (Ψ _π_; %), 0.5SP=1.04±0.01, 2SP=1.13±0.01, 5SP=1.20±0.07, 0.5CL=1.13±0.03, 2CL=1.38±0.03, 5CL=1.72±0.08; (E) water consumption (%), 0.5SP=1.73±0.05, 2SP=1.83±0.07, 5SP=2.13±0.24, 0.5CL=1.53±0.02, 2CL=1.21±0.03, 5CL=1.39±0.04; (F) quantum yield (Qy; %), 0.5SP=0.750±0.002, 2SP=0.753±0.003, 5SP=0.729±0.004, 0.5CL=0.747±0.002, 2CL=0.743±0.001, 5CL=0.735±0.002; (G) net photosynthetic rate (*A*_N_; %), 0.5SP=9.34±1.40, 2SP=9.76±0.76, 5SP=10.1±1.80, 0.5CL=9.71±1.32, 2CL=10.16±1.52, 5CL=9.21±1.59; (H) stomatal conductance (*g*_s_; %), 0.5SP=0.35±0.01, 2SP=0.32±0.08, 5SP=0.31±0.09, 0.5CL=0.30±0.08, 2CL=0.26±0.03, 5CL=0.19±0.03; and (I) photosynthetic or instantaneous water-use efficiency (WUE_i_; %), 0.5SP=26.7±2.95, 2SP=30.2±10.6, 5SP=32.8±3.32, 0.5CL=32.6±6.19, 2CL=39.7±5.28, 5CL=49.0±4.47. Mean values ±SE. *n*=6. ‘Homogeneous group’ statistics were calculated through ANOVA and MANOVA, where mean values with asterisks are significantly different according to Tukey’s test at *P*≤0.05. Levels of significance: ***P≤0.001; **P≤0.01; *P≤0.05; and ‘ns’ *P*>0.05.

To further investigate the role of Cl^−^ nutrition on drought resistance and, accordingly, on WD avoidance and tolerance mechanisms, key parameters were quantified in response to increasing Cl^−^ concentrations (0.5, 2, and 5 mM) and were compared with equivalent gradients of SO_4_^2−^ and PO_4_^3−^ (SP) salts. With this aim, we normalized each measurement obtained under WD conditions with respect to the CTR in each nutritional treatment and, in turn, CL was also normalized to SP plants to show the percentage of improvement induced by Cl^−^ during WD. In Franco-Navarro *et al.* (2016) and Rosales *et al.* (2020), under well-watered conditions, we reported a positive growth response to increasing Cl^−^ treatments, beyond 1 mM Cl^−^. In this work, after 26 d under sustained WD, both 2 mM and 5 mM Cl^−^ treatments significantly induced plant growth and leaf area when compared with SP plants (Fig. 6A, B). The Cl^−^-dependent growth improvement during WD was consistent with higher values of key water parameters such as RWC, Ψ _π_, and water consumption (Fig. 6C–E), which also improved the photosynthetic performance and WUE (Fig. 6F–I). Furthermore, similar experiments in other crop model plants such as tomato confirmed the improvement of growth, RWC, and WUE_i_ during WD by the application of 5 mM Cl^−^ (Supplementary Fig. S6). Therefore, these results confirmed the beneficial effect of macronutrient Cl^−^ nutrition on drought resistance through improvement of plant growth, water balance, gas exchange, and photosynthetic parameters under WD conditions.

## Discussion

In agriculture, Cl^−^ has been frequently considered a harmful anion rather than a plant nutrient due to its toxicity under salt stress conditions and the widespread belief that Cl^−^ impairs NO_3_^−^ nutrition. Recently, we have defined Cl^−^ as a beneficial macronutrient for higher plants with important roles in plant development, water relations, photosynthetic performance, as well as in WUE and NUE (Franco-Navarro *et al*., 2016, 2019; Colmenero-Flores *et al.*, 2019; Rosales *et al.*, 2020). We found that Cl^−^ applied at macronutrient levels reduced *g*_s_ without a concomitant reduction of *A*_N_, which was caused by a compensatory improvement of *g*_m_, enhancing the WUE_i_ of the plant (Franco-Navarro *et al.*, 2019; Raven, 2020). These findings led us to hypothesize that these new Cl^−^-dependent biological functions may affect physiological responses to WD that could improve drought resistance in higher plants.

### Cl^−^ nutrition reduces negative effects of water deficit on plant growth

We observed that the WD treatment significantly reduced plant growth under the three nutritional treatments studied (Fig. 1; Supplementary Fig. S2). However, CL plants grew better (higher total, leaf, and root biomass, and greater leaf area, number, and SLA) during WD, indicating that Cl^−^ induced physiological responses that improve drought resistance. In the agronomic context, some studies have shown that Cl^−^ fertilization (and/or its accompanying cations) could stimulate crop yield (reviewed in Xu *et al.*, 2000), but no information is available regarding its benefits in crops resistance to drought or the physiological mechanisms involved in these responses. The application of mild salt stress (allowing the accumulation of high levels of saline ions in leaf tissues) can improve the water status of plants subsequently subjected to drought in citrus (Pérez-Pérez *et al.*, 2007; Colmenero-Flores *et al.*, 2020), wild barley (Ahmed *et al.*, 2013), and in the xerophyte *Zygophyllum xanthoxylum* (Ma *et al.*, 2012). However, the benefit specifically due to Cl^−^ accumulation had not been clearly established. We found that WD specifically induced an increase in the concentration of Cl^−^ in leaf tissues of CL and SP plants, whereas the NO_3_^−^ concentration showed strong reductions, and no changes were observed in the SO_4_^2−^ concentration (Table 1). This leads to the proposition that plants not only prevent deleterious effects of WD through the accumulation of Cl^−^, but additionally specifically promote Cl^−^ accumulation in response to drought stress (Table 1). Previous observations correlated the accumulation of Cl^−^ in leaves by water shortage with an improvement of the osmotic adjustment in a drought-resistant tomato cultivar (Sánchez-Rodríguez *et al.*, 2010), papaya (Mahouachi *et al.*, 2006), faba bean (Shabala *et al.*, 2000), and in the xerophytic Cl^−^-tolerant species *Pugionium cornutum* (Cui *et al.*, 2020).

As we previously described in Franco-Navarro *et al.* (2016), when water is abundant in the soil, NO_3_^−^-treated plants exhibited the highest growth because of the extra nitrogen fertilization that improves CO_2_ assimilation (Supplementary Fig. S3). However, WD strongly affected the growth of N plants, which exhibited the highest reduction of dry biomass at both the leaf and root levels, as well as the area and number of leaves (Fig. 1B). In contrast to the Cl^−^ accumulation induced by WD in CL and SP plants, WD strongly decreased Cl^−^, NO_3_^−^, SO_4_^2−^, and PO_4_^3−^ in N plants, suggesting a reduced uptake and/or root to shoot translocation of nutrients. This could be due to a more severe restriction of the transpiration rate, as reported in Bista *et al.* (2018). During WD, the leaf NO_3_^−^ content drastically decreased in N plants (~76%) compared with SP and CL treatments (~60 and 35%, respectively; Table 1). This phenomenon could be a consequence of: (i) soil nutrient uptake being more severely impaired in the more stressed N plants; (ii) the stored NO_3_^−^ being used for the synthesis of protecting molecules, including compatible osmolytes (e.g. proline and glycine betaine), non-protein antioxidants (e.g. polyamines), and drought-induced proteins (e.g. LEA, HSPs, and antioxidant enzymes); and (iii) the stored NO_3_^−^ being used in maintaining the plant growth (reviewed in Farooq *et al.*, 2009).

Macronutrient Cl^−^ nutrition has beneficial effects on plant development, water balance, photosynthesis performance, and growth under both well-watered and WD conditions. Cl^−^ is a preferred osmoticum in plants participating in the regulation of cell osmolarity and the electrical charge balance of cations such as K^+^, Ca^2+^, and H^+^ (Flowers, 1988; Broadley *et al.*, 2012). At typical macronutrient concentrations, Cl^−^ represents the dominant inorganic anion in the vacuole, determining more negative osmotic potential and, consequently, higher turgor of plant tissues (Franco-Navarro *et al.*, 2016). Consistently, Cl^−^ stimulates the tonoplast ATPase (Sze, 1985), which induces higher ion compartmentalization in the vacuole, causing higher turgor and, in consequence, increasing the cell growth and water storage capacity of plant cells (reviewed in Colmenero-Flores *et al.*, 2019). Because Cl^−^ is not assimilated in anabolic processes and due to its uncommon physical properties, Cl^−^ becomes a major anion to favour cell water retention, which is crucial for acclimation of plants to WD and cell dehydration. When water is scarce in the soil, Cl^−^ accumulated in leaves at higher concentrations than other anions such as NO_3_^−^, SO_4_^2−^, PO_4_^3−^ (Table 1), and malate (Franco-Navarro *et al.*, 2016), which is consistent with the more negative Ψ _π_ and higher Ψ _p_ of CL plants (Fig. 4B). This indicates that CL plants, showing larger leaf cells with higher osmotic ability, have higher capacity to accumulate water in photosynthetic tissues, which is confirmed by the higher water content, RWC, and succulence of leaves of tobacco plants under WD (Fig. 3A–C). When soil water content decreases, plant cells osmotically adjust to avoid the loss of water. This drought tolerance trait, described as a dehydration avoidance mechanism, is carried out by accumulating osmolytes and/or cell wall hardening (Verslues *et al.*, 2006). Under WD, the contribution of Cl^−^ to the osmotic potential in CL plants was 7.2 times higher than that of SO_4_^2−^ and PO_4_^3−^ in SP plants, and 14.6 times higher than that of NO_3_^−^ in N plants (Supplementary Table S3). Thus, Cl^−^ accumulation in plant tissues arises as a relevant component of the plant osmotic adjustment, which improves osmoregulation, water content, and turgor of plant cells and tissues, favouring cell dehydration avoidance and, therefore, improving WD tolerance.

Leaf patch-clamp pressure probes clearly showed the participation of Cl^−^ in the ability of plants to maintain turgor during the WD treatment (Fig. 4A). Variations between nutritional treatments in the diurnal amplitude of *P*_p_ were probably due to differences in the initial clamp pressure, leaf thickness, or compressibility variations (Zimmermann *et al.*, 2008). Nitrate-treated plants (N) were the first to show turgor loss and also showed inverse *P*_p_ curves after the stress recovery (Fig. 4), indicating lower stress resistance and irreversible leaf tissue damage (Bramley *et al.*, 2013; Fernández *et al.*, 2011; Ehrenberger *et al.*, 2012). SP plants also showed significant turgor loss and partial recovery after re-watering. Interestingly, CL plants were able to maintain the highest turgor during water restriction and managed to fully recover the daily *P*_p_ curves after re-watering. Accordingly, Qy measurements showed that CL plants displayed the lowest symptoms of stress before and after the stress recovery (Fig. 4A). Therefore, turgor maintenance and lower stress symptoms during WD corroborates the role of Cl^−^ nutrition at macronutrient levels in improving drought tolerance in higher plants.

### Cl^−^ nutrition improves water relations and water-use efficiency

Applying Cl^−^ increased WUE not only in well-watered plants (Franco-Navarro *et al.*, 2019; Figs 2A, 5C), but also when soil water availability is limited (Figs 2B, 5C). Under well-watered conditions, Cl^−^ nutrition improved WUE by decreasing transpirational water loss caused by reducing stomatal density and *g*_s_. Photosynthesis is not negatively affected because Cl^−^ induces in turn a higher *g*_m_ (Franco-Navarro *et al.*, 2019; Maron *et al.*, 2019). When WD is imposed, CL plants present a whole array of physiological advantages: (i) better WD avoidance due to more efficient water use (Figs 2, 5C); (ii) higher water content in photosynthetic tissues (Fig. 3A, B); (iii) better cell dehydration avoidance because of a higher osmoregulatory capacity (Fig. 3B), allowing higher turgor maintenance (Fig. 4A, B); and (iv) better cell dehydration tolerance leading to higher protection of cell macromolecules, including the photosynthetic machinery (Figs 4–6). Thus, enhanced water status in Cl^−^-treated plants is expected to further prevent strong stomatal closure, allowing higher transpiration, as observed in CL plants exhibiting higher *g*_s_ (Fig. 5A), and higher water consumption (Fig. 6E), than SP and N plants under WD conditions. In turn, higher *g*_s_, together with the greater integrity of the photosynthetic machinery (Fig. 5D), determines higher photosynthetic efficiency in CL plants (Figs 5B, 6G), allowing higher biomass production under WD conditions (Figs 1, 2, 6A). Therefore, Cl^−^-dependent mechanisms responsible for the higher WUE in well-irrigated plants (lower *g*_s_ and water consumption compensated by a higher *g*_m_, maintaining similar *A*_N_; Figs 2C, 5A) differ from those responsible for the higher WUE in WD-stressed plants (greater *g*_s_ and water consumption allowing higher *A*_N_ and biomass production; Figs 2D, 5A). This is important since photosynthesis is greatly affected by WD, causing partial stomatal closure that leads to a decrease in the CO_2_ availability (Chaves *et al.*, 2009).

An additional advantage of CL plants under WD conditions could be the greater SLA, determining higher leaf area for a given leaf biomass than SP and N plants (Fig. 1B). Drought commonly decreases SLA as a result of more compactly packed leaf cells, leading to CO_2_ diffusion limitations and lower photosynthetic rates (Niinemets, 1999). Surprisingly, the CL treatment not only determined higher SLA values relative to the SP and N treatments, but also determined higher SLA values in WD than in control conditions, meaning more expanded leaves for a similar biomass. This observation correlates with CL plants having more water (succulence) per cm^2^ than SP and N plants. Thus, CL plants with higher SLA have more light-capturing surface per unit of biomass invested (Fig. 1B), which might improve higher assimilation and respiration rates (Poorter and Bongers, 2006).

### Potential benefits of Cl^−^ nutrition in agriculture

Under drought, small morphological changes in leaves can considerably increase WUE, leading to a competitive advantage in crop yield (reviewed in Flexas *et al.*, 2016). Our findings are therefore particularly relevant because of the possibility that agricultural practices ensuring adequate Cl^−^ management might increase crop WUE. The most frequent leaf Cl^−^ concentrations from 670 species belonging to 138 families of land plants collected from their natural habitats were ~5 mg g^–1^ DW (Watanabe *et al.*, 2007), which is well above the critical requirement as a micronutrient, but below the beneficial Cl^−^ content requirement to induce WD protection in tobacco plants (20–50 mg g^–1^ DW; Fig. 6). Therefore, plants might frequently benefit from Cl^−^ fertilization in different environments. To that end, Cl^−^ levels present in agricultural soils, together with those required in different plant species to induce beneficial effects, should be determined to improve agronomic Cl^−^ management. In a complementary study in tomato plants, we observed that Cl^−^ also stimulated plant growth during WD, consistent with a better water status and WUE_i_ (Supplementary Fig. S6). Therefore, we can expect that the benefits of Cl^−^ fertilization could be extended to other crop species.

Nitrogen is the most limiting nutrient for plant growth, with NO_3_^−^ as a major nitrogen source regulating many physiological processes (reviewed in Krapp *et al.*, 2014). However, additional NO_3_^−^ fertilization (in N plants) increased sensitivity to WD, with a strong reduction of growth and WUE compared with CL and SP plants (Figs 1, 2). The N plants had more and larger leaves (Supplementary Fig. S3), with greater *g*_s_ and higher requirement of water consumption (Fig. 5; Supplementary Fig. S1A). Nitrate is the most widely used fertilizer in agriculture, as well as a source of environmental pollution. The similarity between Cl^−^ and NO_3_^−^ molecules determines functional overlap (sharing membrane transport mechanisms and functions such as counteranion and cell osmoregulation), which implies strong dynamic interactions between the two monovalent anions in plants (Wege *et al.*, 2017; Colmenero-Flores *et al.*, 2019). We propose that according to the environmental conditions or to crop water management (e.g. if we anticipate a deficit irrigation period), it could be useful to adjust optimal NO_3_^−^:Cl^−^ ratios to adequately balance growth versus drought acclimatization abilities of plants. For example, substituting part of the NO_3_^−^ by Cl^−^ in the fertilizer can reduce the release of nitrogen into the environment, increasing the crop NUE (as we have already shown in Rosales *et al.*, 2020), and making plants less sensitive to deficit irrigation or drought under conditions in which the bottleneck for crop production is not nitrogen but water availability.

**Fig. 7.**
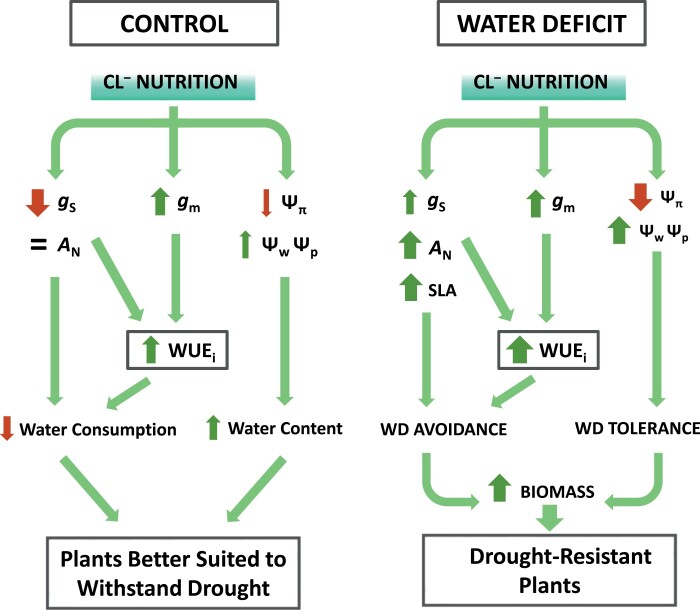
Integrative model of macronutrient Cl^−^ nutrition effects during control and WD conditions. This model integrates the effects of Cl^−^ nutrition (compared with SP and N plants) on growth, water balance, and water-use efficiency parameters in tobacco plants subjected to both control and WD conditions, from results obtained in this work and in combination with those obtained in Franco-Navarro *et al*. (2016, 2019). Up and down arrows represent significantly increased and reduced responses, respectively. No change is represented by the equals sign. The intensity of the responses is shown by the thickness of arrows (thicker line represents stronger response).

In conclusion, we have demonstrated for the first time that Cl^−^ nutrition at macronutrient levels improves drought resistance as a result of the simultaneous occurrence of WD avoidance and tolerance mechanisms (Fig. 7). This work is in line of with our definition of Cl^−^ as a beneficial macronutrient due to its ability to improve plant development, tissue water balance, whole-plant water relations, and photosynthesis performance in well-watered plants, which results in more efficient use of water (WUE), nitrogen (NUE), and CO_2_. Thus, our results showed that Cl^−^ treatments (2–5 mM) reduced stress symptoms and allowed continued growth in tobacco plants during WD as a result of two main mechanisms (Fig. 7): (i) improved osmoregulation, allowing higher turgor and water content in photosynthetic tissues, which favours cell dehydration avoidance and WD tolerance; and (ii) improved WUE, allowing better WD avoidance, as a result of higher *g*_s_ and water consumption that increases *A*_N_ and plant biomass. Therefore, implementing agronomic practices that ensure beneficial Cl^−^ levels and optimal Cl^−^:NO_3_^−^ ratios in the field is expected to improve crop WUE and drought resistance, as well as to reduce the use of nitrogen fertilizers and nitrate pollution, promoting a more sustainable and resilient agriculture.

## Supplementary data

The following supplementary data are available at *JXB* online.

Fig. S1. Time course of substrate water loss.

Fig. S2. Images showing effects of Cl^−^ nutrition and WD on plant growth.

Fig. S3. Effect of Cl^−^ nutrition and sustained WD on plant and leaf growth.

Fig. S4. Relationship between total biomass and water consumption.

Fig. S5. Relationship between *A*_N_ and RWC.

Fig. S6. Effect of Cl^−^ nutrition and sustained WD on leaf biomass, RWC, and WUE_i_ in tomato plants.

Table S1. List of experiments conducted between 2010 and 2018 to characterize the role of Cl^−^ nutrition in different physiological processes.

Table S2. Anion content in leaves subjected to different nutritional and irrigation treatments.

Table S3. Osmotic potential calculated from ion concentration measured in mature leaves.

erab143_suppl_Supplementary_Figures_and_Tables

## Data Availability

Data supporting the findings of this study are available within the paper and within its supplementary data published online. Further information may be obtained from the corresponding author.

## Abbreviations

ABA: abscisic acid
*A*_N_: net photosynthetic rate
Cl^−^: chloride
CTR: control
DAS: days after sowing
*g*_m_: mesophyll diffusion conductance to CO_2_
*g*_s_: stomatal conductance
NO_3_^−^: nitrate
NUE: nitrogen-use efficiency
PO_4_^3−^: phosphate
*P*_p_: patch output pressure
Qy: PSII quantum yield
RWC: relative water content
SLA: specific leaf area
SO_4_^2−^: sulfate
WD: water deficit
WUE: water-use efficiency
WUE_i_: intrinsic water-use efficiency
Ψ _π_: osmotic potential
Ψ _p_: turgor potential
Ψ _W_: water potential
