# Supplementary material for: Chloride nutrition improves drought resistance by enhancing water deficit avoidance and tolerance mechanisms
Source: J Exp Bot. 2021 Mar 30;72(14):5246–61. doi: 10.1093/jxb/erab143 (PMC8272566; doi:10.1093/jxb/erab143)
Supplement: erab143_suppl_Supplementary_Figures_and_Tables [file erab143_suppl_supplementary_figures_and_tables.pdf]

Macronutrient chloride nutrition improves drought resistance by enhancing water deficit avoidance and tolerance mechanisms

Juan D. Franco-Navarro[1], Pablo Díaz-Rueda[1], Carlos M. Rivero-Núñez[1], Javier Brumós[2], Alfredo Rubio-Casal[3], Alfonso de Cires[3], José M. Colmenero-Flores[1,4]* & Miguel A. Rosales[1,4]*

[1]Group of Plant Ion and Water Regulation, Instituto de Recursos Naturales y Agrobiología, Consejo Superior de Investigaciones Científicas (CSIC), 41012-Seville, Spain.

[2]Instituto Valenciano de Investigaciones Agrarias, Centro de Genómica, Moncada, 46113-Valencia, Spain

[3]Departamento de Biología Vegetal y Ecología, Facultad de Biología, Universidad de Sevilla, 41012-Seville, Spain.

[4]Laboratory of Plant Molecular Ecophysiology, Instituto de Recursos Naturales y Agrobiología, Consejo Superior de Investigaciones Científicas (CSIC), 41012-Seville, Spain.

***Corresponding authors details:** Miguel A. Rosales, mrosales@irnas.csic.es, and José M. Colmenero-Flores, chemacf@irnase.csic.es

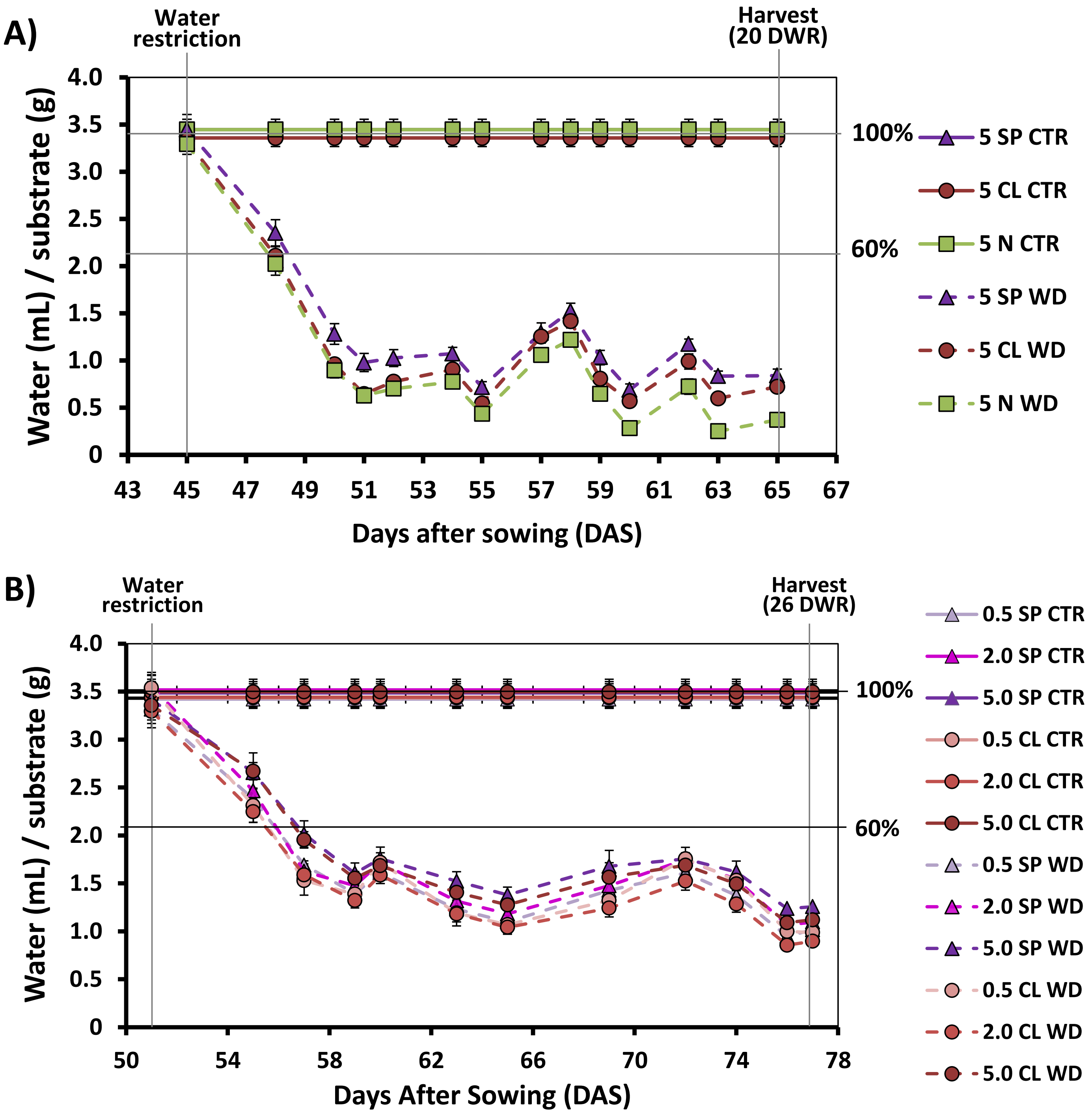

Figure S1. Time course of substrate water loss. Plants were alternatively treated with the following salt supplements in two different experiments: (A) 5 mM chloride salts (CL); 5 mM nitrate salts (N); and a mixture of sulphate + phosphate salts (SP) containing the same cationic balance as in the CL and N treatments. Tobacco plants were grown in pots for 30 days under those nutritional treatments. Afterwards, two irrigation treatments were imposed at 45 days after sowing (DAS): 100% field capacity (3.5 mL g^{-1} substrate; CTR, control), and 60% of field capacity (2.1 mL g^{-1} substrate; WD, water deficit). (B) Increasing concentrations of 0.5 mM, 2 mM and 5 mM Cl^{-} or SO_4^{2-} + PO_4^{3-} salts were applied to the basal solution, containing the same cationic balance in both treatments. The graphic shows the average water content per pot. Samples were taken before irrigation at the indicated times. Mean values ± SE, n = 4-6. Days of Water Restriction (DWR).

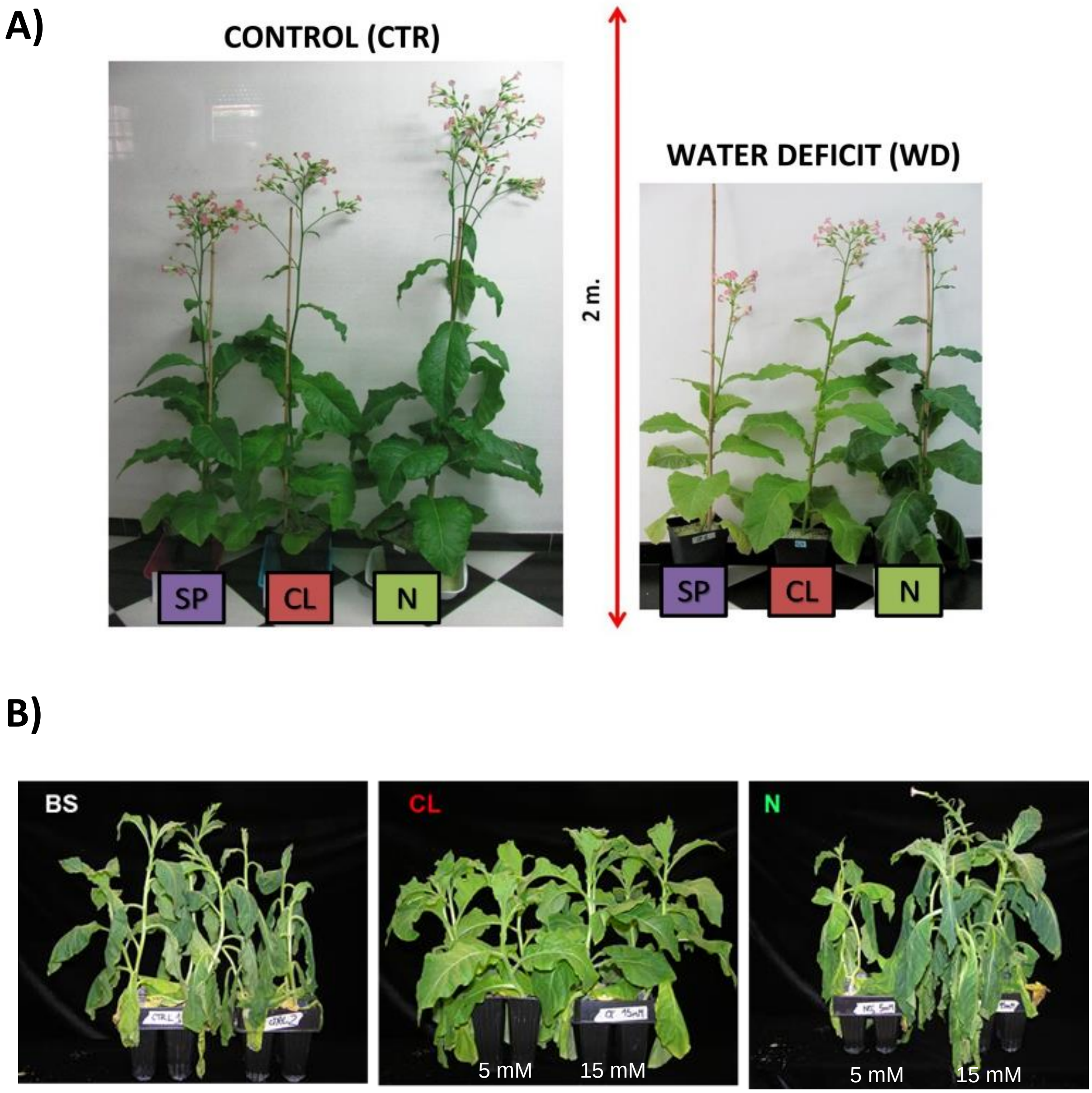

Figure S2. Images showing effects of Cl^- nutrition and water deficit on plant growth. Plants were watered with a basal solution (BS) and alternatively treated with three nutritional treatment: 5 mM chloride salts (CL); 5 mM nitrate salts (N); and a mixture of sulphate + phosphate salts (SP) containing the same cationic balance as in the CL and N treatments (A). Additionally, plants watered with BS were subjected to 5 and 15 mM chloride and nitrate salts (B). Figure shows comparative images of tobacco plants subjected to control (CTR; 100% field capacity) and water deficit (WD; 60% field capacity) treatments sustained for 20 days (A), and after 5 days of complete water deprivation (B).

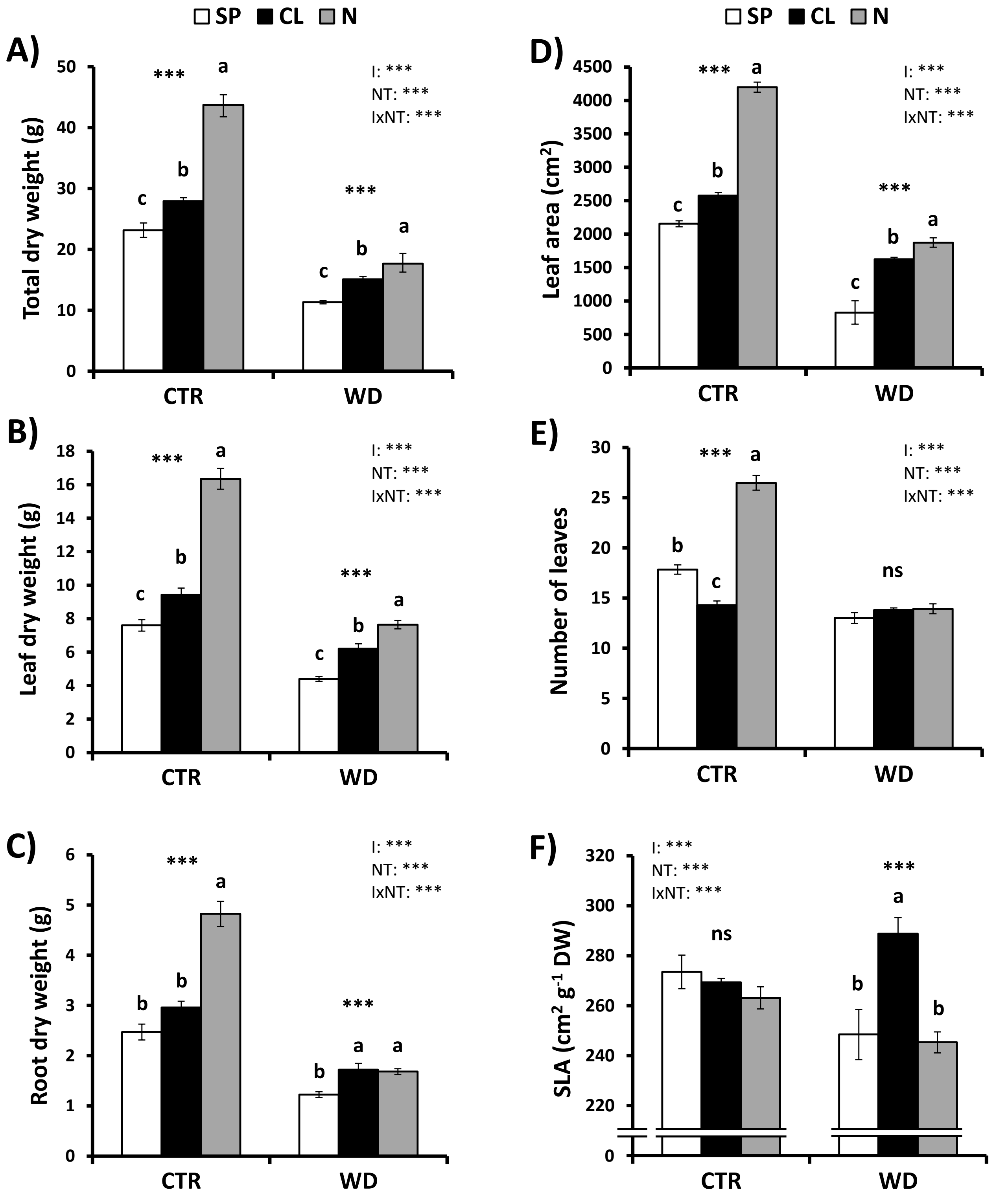

Figure S3. Effect of Cl⁻ nutrition and sustained water deficit on plant and leaf growth. Plants were alternatively treated with (i) three nutritional treatments (NT): 5 mM chloride salts (CL); 5 mM nitrate salts (N); and a mixture of sulphate + phosphate salts (SP) containing the same cationic balance as in the CL and N treatments; and (ii) two irrigation treatments (I) were also applied: 100% field capacity (CTR, control) and 60% field capacity (WD, water deficit). (A) Effect on total dry biomass. (B) Effect on leaf dry biomass. (C) Effect on root dry biomass. (D) Effect on total leaf area. (E) Effect on total number of leaves. (F) Effect on specific leaf area (SLA). Mean values ± SE, *n* = 6. 'Homogeneous group' statistics were calculated through ANOVA, where mean values with different letters are significantly different according to Tukey's test at $P \leq 0.05$. Levels of significance: $^{***}P \leq 0.001$; and 'ns' $P > 0.05$.

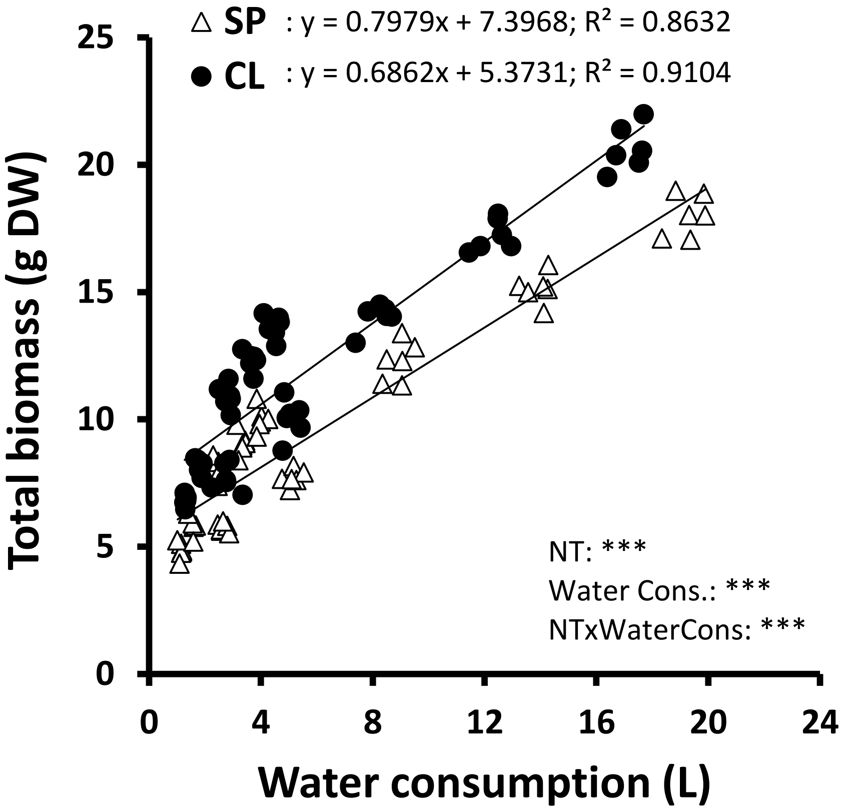

Figure S4. Relationship between total biomass and water consumption. Plants were alternatively treated with (i) two nutritional treatments (NT): 5 mM chloride salts (CL, circles) and a mixture of sulphate + phosphate salts (SP, triangles) containing the same cationic balance as in the CL treatment; and (ii) two irrigation treatments were also applied: 100% field capacity (CTR, control) and 60% field capacity (WD, water deficit). The regression line for each SP and CL pools is shown in the figure (including CTR and WD values), where *P*-values and ANCOVA to compare regression slopes are shown. Levels of significance: ***$P \leq 0.001$.

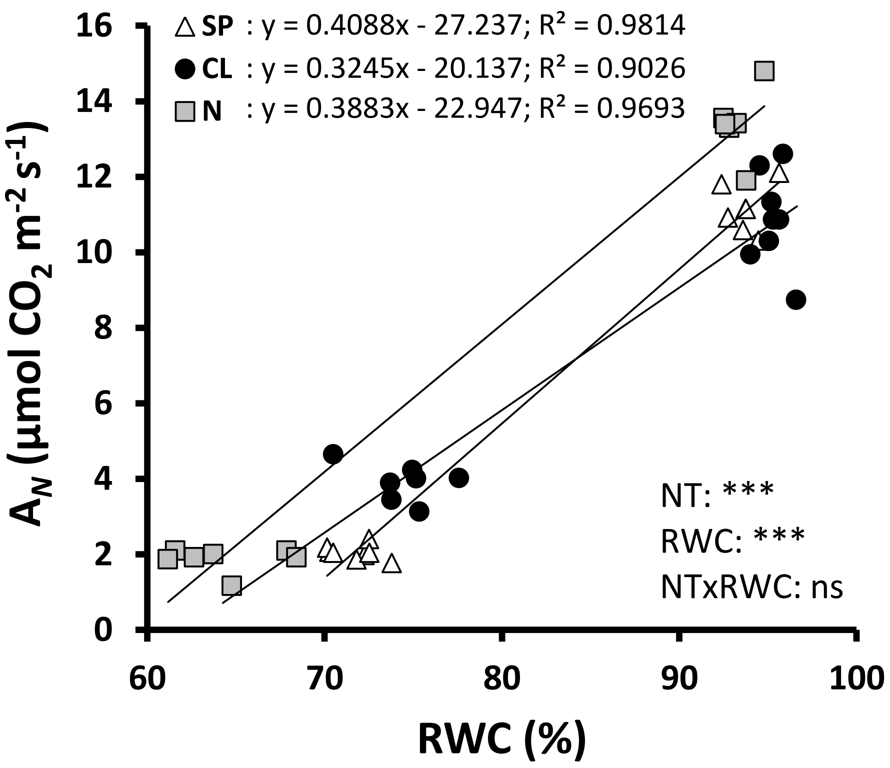

Figure S5. Relationship between net photosynthetic rate (A_N) and relative water content (RWC). Plants were alternatively treated with (i) three nutritional treatments (NT): 5 mM chloride salts (CL, black circles); 5 mM nitrate salts (N, grey squares); and a mixture of sulphate + phosphate salts (SP, white triangles) containing the same cationic balance as in the CL and N treatments; and (ii) two irrigation treatments were also applied: 100% field capacity (CTR, control) and 60% field capacity (WD, water deficit). The regression line for each SP, CL and N pools is shown in the figure, where *P*-values and ANCOVA to compare regression slopes are shown. Levels of significance: ****P* ≤ 0.001; and 'ns' *P* > 0.05.

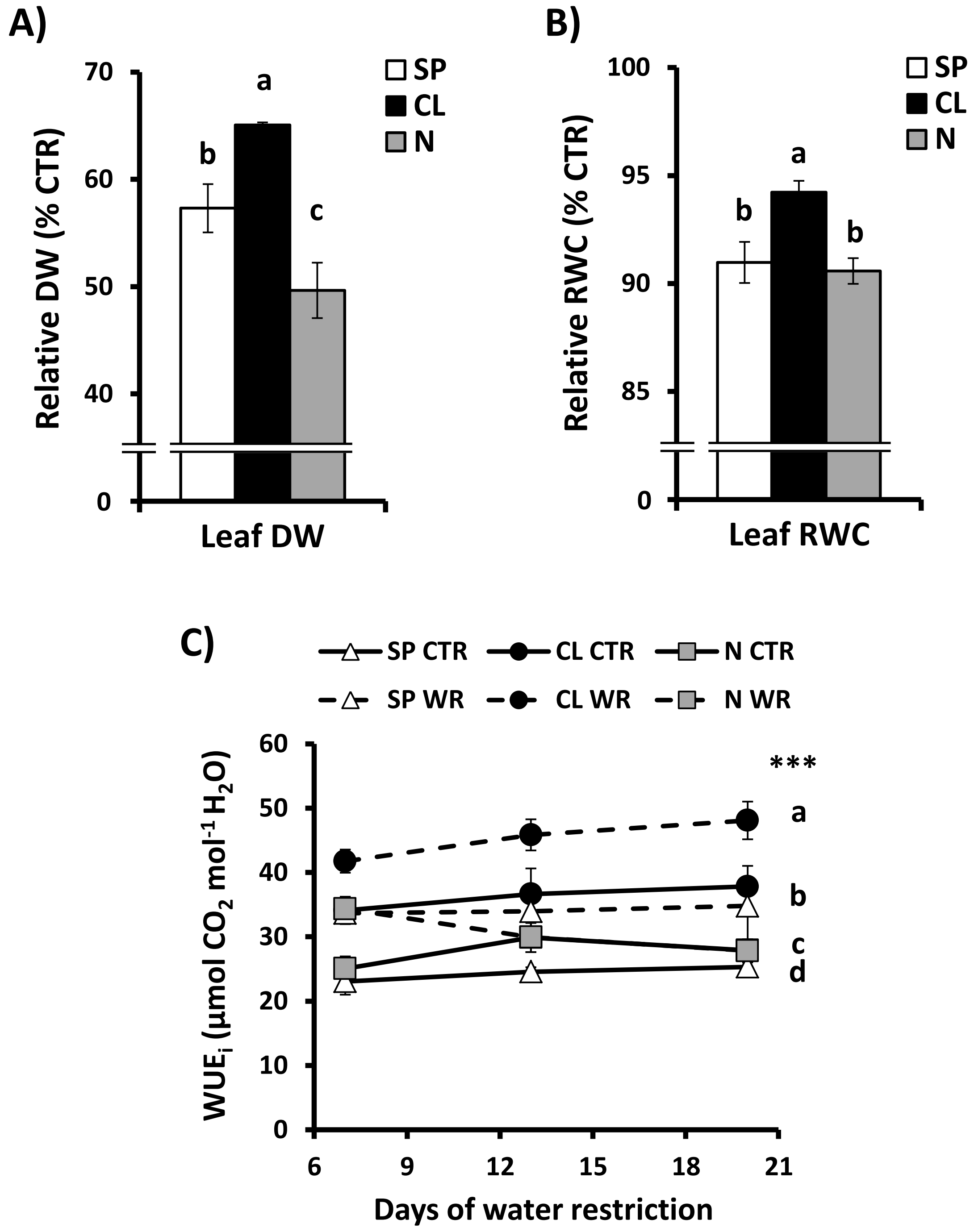

Figure S6. Effect of Cl⁻ nutrition and sustained water deficit on leaf biomass, RWC and WUE$_i$ in tomato plants. Tomato plants were alternatively treated with (i) three nutritional treatment: 5 mM chloride salts (CL); 5 mM nitrate salts (N); and a mixture of sulphate + phosphate salts (SP) containing the same cationic balance as in the CL and N treatments; and (ii) two irrigation treatments were also applied: 100% field capacity (CTR, control) and 60% field capacity (WD, water deficit). (A) Effect on leaf dry weight (%; DW) in WD plants normalized to CTR plants. (B) Effect on leaf relative water content (%; RWC) in WD plants normalized to CTR plants. (C) Effect on integrated water-use efficiency (WUE) in plants subjected to CTR and WD treatments. Mean values ± SE, n = 6. 'Homogeneous group' statistics were calculated through ANOVA, where mean values with different letters are significantly different according to Tukey's test at $P \leq 0.05$. Levels of significance: ***$P \leq 0.001$.

Table S1. List of experiments conducted between 2010-2018 to characterize the role of Cl^- nutrition on different physiological processes.

#	Date	Treatments	Objectives	Publication
3	MAY-JUL 2010	BS, N, CL; 5 mM	Tobacco. Water relations.	Franco-Navarro et al. (2016)
4	MAY-JUL 2010	BS, N, CL; 5 mM	Tobacco. Water relations.	Franco-Navarro et al. (2016)
**5**	**MAY-AUG 2010**	**BS, N, CL; 1, 2.5, 5 mM; CTR, WD**	**Tobacco. WD.**	**This work**<br>Franco-Navarro et al. (2016)
6	JUL-SEP 2010	BS, N, CL; 5 mM	Tobacco. Water relations.	Franco-Navarro et al. (2016)
7	NOV-DIC 2010	BS, N, CL; 5 mM	Tobacco. Water relations.	Franco-Navarro et al. (2016)
8	DIC 2010-MAR 2011	BS, N, CL; 1, 3, 5 mM	Tobacco. Water relations.	Franco-Navarro et al. (2016)
9	NOV 2010-JAN 2011	BS, N, CL; 5 mM	Tobacco. Water relations.	Franco-Navarro et al. (2016)
10	MAR-JUN 2011	BS, N, CL; 5 mM	Tobacco. Cell analysis.	Franco-Navarro et al. (2019)
**13**	**APR-JUN 2011**	**BS, N, CL; 5 mM; CTR, WD**	**Tobacco. WD.**	**This work**
**15**	**JUN-AUG 2011**	**BS, N, CL; 5 mM; CTR, WD**	**Tobacco. WD.**	**This work**
17	JUL-OCT 2011	BS, N, CL; 5 mM; ABA	Tobacco. Leaf cells.	Franco-Navarro et al. (2019)
20	DEC 2011-MAR2012	BS, SP, N, CL; 5 mM	Tobacco.	Franco-Navarro et al. (2016)
**21**	**FEB-APR 2012**	**SP, N, CL; 5 mM; CTR, WD**	**Tobacco. WD.**	**This work**
23	AUG-SEP 2012	SP, N, CL; 0.1 -15 mM	Tobacco. Increasing ratios.	Franco-Navarro et al. (2016)
**24**	**OCT-DEC 2012**	**SP, N, CL; 5 mM; CTR, WD**	**Tobacco. WD.**	**This work**
27	JAN-MAR 2013	SP, N, CL; 5 mM	Tobacco. G_m.	Franco-Navarro et al. (2019)
28	APR-MAY 2013	BS, SP, CL ; 0.15-5 mM	Tobacco. Increasing ratios.	Franco-Navarro et al. (2016)
**29**	**AUG-OCT 2013**	**BS, SP, N, CL; 5 mM; CTR, WD**	**Tobacco. WD.**	**This work**
**31**	**MAY-JUN 2014**	**BS, SP, N, CL; 5 mM; CTR, WD**	**Tobacco. WD**	**This work**
**32**	**OCT 2014-JAN 2015**	**BS, SP, N, CL; 5 mM; CTR, WD**	**Tobacco. WD.**	**This work**
34	FEB-MAY 2016	SP, N, CL; 5 mM	Tobacco. Microscopy.	Franco-Navarro et al. (2019)
35	APR-JUL 2016	SP, N, CL; 5 mM	Tobacco. Microscopy.	Franco-Navarro et al. (2019)
36	SEPT-DEC 2016	SP, N, CL; 5 mM	Crops. NUE.	Rosales et al. (2020)
**37**	**SEPT-DEC 2016**	**Ratios N:CL:SP; CTR, WD**	**Tobacco. NUE. WD.**	**This work**<br>Rosales et al. (2020)
38	OCT 2016-FEB 2017	SP, N, CL; 5 mM	Crops. Xylem sap. NUE.	Rosales *et al.* (2020)
**39**	**JAN-APR 2017**	**Ratios N:CL:SP**	**TOBACCO. NUE. WD.**	**This work**
40	FEB-JUN 2017	SP, N, CL; 5 mM	15 different species.	Rosales *et al.* (2020)
**41**	**DEC-MAR 2017**	**Ratios N:CL:SP; CTR, WD**	**Tobacco. Crops. WD. NUE.**	**This work**<br>Rosales *et al.* (2020)
42	JUN-JUL 2017	SP, N, CL; 5 mM	Crops. Young plants.	Rosales et al. (2020)
**45**	**AUG-OCT 2018**	**SP, N, CL; 5 mM**	**Tobacco. WD. Time lapse.**	**This work**

Treatments: BS, basal solution; SP, sulphate+phosphate; N, nitrate; CL, chloride; CTR, well-irrigated; WD, water deficit. Yellow rows represent experiments used for figures and tables presented in the present article and biological repetitions. Papers published: Franco-Navarro et al. (2016; J. Exp. Bot. 67:873; blue rows); Franco-Navarro et al. (2019; Plant J. 99:815; red rows), and Rosales et al. (2020; Front. Plant Sci. 11:442; green rows).

Table S2. Anion content (mg g^{-1} DW) in leaves subjected to different nutritional and irrigation treatments.

	Cl^- (mg g^{-1} DW)			NO_3^- (mg g^{-1} DW)			PO_4^{3-} (mg g^{-1} DW)			SO_4^{2-} (mg g^{-1} DW)		
	**CTR**	**WD**	***P***	**CTR**	**WD**	***P***	**CTR**	**WD**	***P***	**CTR**	**WD**	***P***
**SP**	0.41 ± 0.03 b	0.88 ± 0.23 b	*	7.26 ± 2.06 b	2.96 ± 0.79 b	**	16.9 ± 1.63 **a**	12.8 ± 1.50 **a**	ns	37.2 ± 2.27 **a**	28.7 ± 3.79 **a**	ns
**CL**	55.7 ± 2.01 **a**	49.3 ± 1.33 **a**	*	3.38 ± 0.85 b	2.21 ± 0.64 b	ns	9.77 ± 0.81 b	9.72 ± 0.33 b	ns	17.0 ± 3.61 b	12.4 ± 0.42 b	ns
**N**	0.45 ± 0.07 b	0.38 ± 0.04 b	ns	56.4 ± 7.70 **a**	13.8 ± 1.64 **a**	**	10.9 ± 0.33 b	8.76 ± 0.23 b	***	18.9 ± 4.33 b	16.9 ± 1.18 b	ns
P	***	***		***	**		***	***		***	***	
**I**	**			***			*			*		
**NT**	***			***			***			***		
**I x NT**	***			***			ns			ns		

Nutritional treatment (NT) consisted of a basal nutrient solution supplemented with 5 mM chloride (CL), 5 mM nitrate (N) or the sulphate + phosphate (SP) salt mixture containing the same cationic balance as in the CL and N treatments. Irrigation treatment (I) consisted on a control treatment of well-watered plants (CTR; 100% field capacity), and sustained water deficit (WD; 60% field capacity) treatments. Mean values ± SE, n = 6. Levels of significance: $P \leq 0.001$ (***), $P \leq 0.01$ (**), $P \leq 0.05$ (*), and $P > 0.05$ ('ns', not significant). "Homogeneous group" statistics was calculated through ANOVA and MANOVA tests, where mean values with different letters are significantly different according to Tukey's test.

Table S3. Osmotic potential calculated from ion concentration measured in mature leaves.

**CTR**	**SP**			**CL**			**N**		
	**mM**	***-MPa***	**%**	**mM**	***-MPa***	**%**	**mM**	***-MPa***	**%**
**K^+**	188.23	-0.4847	53.5	173.39	-0.4465	39.9	185.95	-0.4788	50.9
**Ca^{2+}**	49.66	-0.1284	13.7	51.48	-0.1284	11.5	53.31	-0.1330	14.1
**Mg^{2+}**	56.36	-0.2203	25.6	53.53	-0.2203	19.7	54.75	-0.2253	23.9
**Cl^-**	1.02	-0.0029	0.3	106.51	-0.3004	**26.8**	1.01	-0.0028	0.3
**NO_3^-**	6.71	-0.0108	1.2	2.48	-0.0040	0.4	46.88	-0.0757	**8.0**
**SO_4^{2-}**	33.46	-0.0349	**3.8**	12.02	-0.0125	1.1	15.51	-0.0162	1.7
**PO_4^{3-}**	15.66	-0.0165	**1.8**	6.97	-0.0073	0.7	9.02	-0.0095	1.0
**Ψπ**		-0.9056			-1.1195			-0.9413	

**WD**	**SP**			**CL**			**N**		
	**mM**	***-MPa***	**%**	**mM**	***-MPa***	**%**	**mM**	***-MPa***	**%**
**K^+**	221.16	-0.5695	51.7	194.96	-0.5020	39.4	187.96	-0.4840	48.3
**Ca^{2+}**	71.18	-0.1776	16.1	70.33	-0.1755	13.8	70.19	-0.1751	17.5
**Mg^{2+}**	71.91	-0.2959	26.9	57.69	-0.2374	18.6	70.29	-0.2892	28.9
**Cl^-**	2.56	-0.0072	0.7	118.95	-0.3355	**26.3**	1.05	-0.0030	0.3
**NO_3^-**	3.20	-0.0052	0.5	2.00	-0.0032	0.3	14.20	-0.0229	**2.3**
**SO_4^{2-}**	30.75	-0.0320	**2.9**	11.05	-0.0115	0.9	17.15	-0.0179	1.8
**PO_4^{3-}**	13.84	-0.0146	**1.3**	8.76	-0.0092	0.7	9.01	-0.0095	0.9
**Ψπ**		-1.1020			-1.2744			-1.0016	

Nutritional treatment (NT) consisted of a basal nutrient solution supplemented with 5 mM chloride (CL), 5 mM nitrate (N) or the sulphate + phosphate (SP) salt mixture containing the same cationic balance as in the CL and N treatments. Irrigation treatment consisted on a control treatment of well-watered plants (CTR; 100% field capacity), and sustained water deficit (WD; 60% field capacity) treatments. Osmotic potential (Ψπ) was calculated by Van Hoff's equation from the ion concentration obtained in mature leaves. Mean values, n = 6.
